# Nonconventional Full-Color Luminescent Polyurethanes: Luminescence Mechanism at the Molecular Orbital Level

**DOI:** 10.1021/acsmaterialslett.4c02100

**Published:** 2024-11-21

**Authors:** Nan Jiang, Ya-Jie Meng, Xin Pu, Chang-Yi Zhu, Shu-Han Tan, Yan-Hong Xu, You-Liang Zhu, Jia-Wei Xu, Martin R. Bryce

**Affiliations:** † Key Laboratory of Preparation and Applications of Environmental Friendly Materials, Key Laboratory of Functional Materials Physics and Chemistry of the Ministry of Education, Jilin Normal University, Changchun, 130103, China; ‡ Ministry-of-Education Key Laboratory of Numerical Simulation of Large-Scale Complex System (NSLSCS) and School of Chemistry and Materials Science, Nanjing Normal University, Nanjing 210023, China; § State Key Laboratory of Supramolecular Structure and Materials, College of Chemistry, Jilin University, Changchun, 130012, China; ∥ Department of Chemistry, Durham University, Durham, DH1 3LE, U.K.

## Abstract

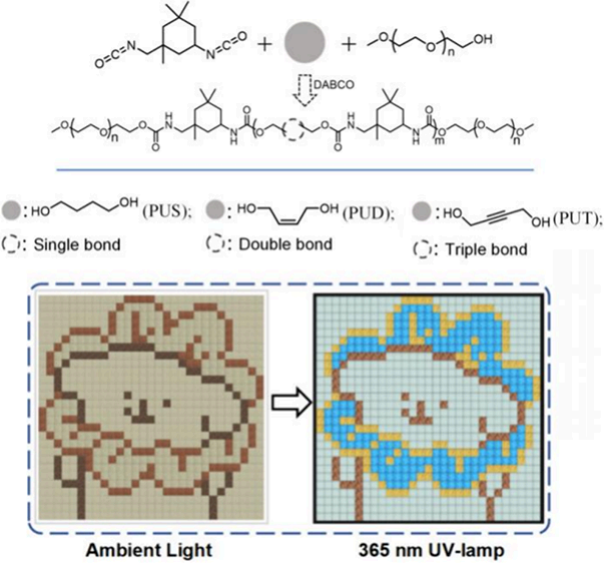

The study of structure–activity relationships is a top priority in the development of nontraditional luminescent materials. In this work, nonconjugated polyurethanes (PUs) with full-color emission (red, green, and blue) are easily obtained by control of the diol monomer structure and the polymerization conditions. Selected diol monomers introduced single, double, or triple bond repeating units into the main chain of the PUs, in order to understand how unsaturated bonds and H-bonds affect their luminescence from a molecular orbital viewpoint. Detailed experimental and theoretical results show that the PUs have different temperature-dependent behaviors related to the interplay of H-bonding, through-space n−π interactions, and aggregation properties. The potential applications of PUs in colorful displays, covert information transmission, and multifunctional bioimaging have been verified. This work provides a new general protocol for the simple preparation of multifunctional nonconventional fluorescent polymers and deepens the understanding of their luminescence mechanisms.

In 1899, the genius inventor Tesla said, “Everything is the light.”^1^ People have never stopped pursuing the origins of light and exploiting luminescent phenomena. Traditional luminescent polymers contain large π-conjugated (hetero)aromatic structures, and through-bond conjugation is the primary source of their emission,^2,3^ in some cases aided by intra- and interchain heteroatom effects.^4^ However, these materials are often complex and expensive to synthesize, require environmentally toxic reagents and catalysts, have limited processability, and are mostly nonbiodegradable, which may significantly restrict their long-term and large-scale applications.^5−7^ However, some nonconjugated luminescent polymers (NCLPs) such as starch, cellulose, and polyacrylate can also emit visible light through noncovalent electronic overlap and delocalization in heteroatom-rich segments.^5−14^ NCLPs with properties such as good biocompatibility, low cost, and good processability are promising materials.^15−17^ A drawback is that they typically emit light of shorter wavelengths (400–500 nm, rarely above 600 nm) and relatively monochromatic light, which greatly limits their application development.^18−20^ Besides, how weak molecular interactions affect the luminescence of NCLPs at the molecular orbital level has always been obscure. Therefore, the simple construction of full-color luminescent NCLPs and in-depth insights into the luminescence mechanism are very timely.

To achieve multicolor luminescence, many studies have relied on the excitation-dependent fluorescence (EDF) of NCLPs, which refers to different emission wavelengths obtained by changing the excitation wavelength.^21−24^ However, this method has several limitations: first, although short-wavelength-excited NCLPs may emit at longer wavelengths, the intensity of the longer-wavelength emissions decreases as the excitation wavelength increases, eventually even approaching zero. Second, many NCLPs do not exhibit EDF characteristics. Third, although EDF may be apparent in the spectral analysis, many commercially available UV lamps emit mixed-wavelength light, unlike lasers or fluorescence spectrometers which typically emit light of a single wavelength. Therefore, in practical applications, changing the excitation wavelength of UV lamps may not result in significant fluorescence changes. Strategies like adjusting through-space interactions between and/or within chains, introducing multiple unconventional chromophores, or changing the relative rigidity and flexibility of the polymer chains have also been used to obtain multicolor NCLPs.^25−27^ Alternatively, the luminescence of NCLPs can be adjusted through heating (including water and air heating).^28−30^ Additionally, due to the abundance of noncovalent interactions, NCLPs are highly susceptible to the influence of the microenvironment, and parameters such as pH can alter their luminescent behavior.^31−33^ However, these external regulatory factors complicate the study of luminescence in NCLPs’ aggregated states.

Recently, a new approach to full-color nonconventional luminescence in poly(maleimide) derivatives, without any aromatics, was based on the bonding modes (C–C and C–N linkages) in the backbone.^26^ We now report a different strategy: namely, intramolecular/intermolecular interactions of polyurethane (PU) chains regulated by introducing single bond (alkane), double bond (alkene), or triple bond (alkyne) repeating units in the backbone lead to full-color emission. Polyurethane is well-known as one of the world’s major plastics.^34^ Different combinations of diisocyanate and diol monomers give PUs with very varied properties and extensive applications in textiles, plastics, electronics, and medical materials.^35−38^ Recent encouraging biodegradability studies make PUs excellent candidates for NCLP developments.^39,40^ In this context, the urethane [−NHC(O)O−] repeat units provide abundant inter/intrachain interactions conducive to aggregation behavior, electronic overlap and delocalization, such as C=O···N–H (dipole–dipole), C=O···C=O (n-π*), O=C···C=O (π–π*), O···O, and hydrogen bonding.^40−42^ Furthermore, the unique soft and hard segment microphase separation structure of PUs gives conformational variability in the aggregated states.^43,44^ Therefore, PUs are ideal templates for tuning multicolor luminescence and studying the aggregation luminescence mechanism.

Nine PU derivatives have now been synthesized by a one-pot reaction of isophorone diisocyanate, poly(ethylene glycol) monomethyl ether, and diol monomers with different central C–C, C=C, or C≡C units (namely, 1,4-butanediol, (*Z*)-2-butene-1,4-diol, and 2-butyne-1,4-diol). The synthetic route is shown in Figure 1, and structural characterization is detailed in Figures S1–S4. Nuclear magnetic resonance (NMR) spectroscopy shows that all the **PUS**, **PUD**, and **PUT** series are well-structured materials (Figures S1–S3). The molecular weight and molecular weight distribution of the PUs determined by gel permeation chromatography (GPC) show that the products have *M*_n_ values in the ranges 2874–2119 (**PUS**), 3870–2190 (**PUD**), and 3668–1282 (**PUT**) (Figure 1 and Table S1). FT-IR spectroscopy (Figure S4) confirmed the presence of a characteristic functional urethane unit. Differential scanning calorimetry (DSC) showed that the introduction of double bonds increased the rigidity of the chain segment and the glass transition temperature (*T*_g_) of the **PUD** series generally increased compared with the **PUS** series. However, it is possible that because the introduction of the alkyne group reduces the forces such as bending or entanglement of the soft segments, the **PUT** series exhibits lower *T*_g_ values. Also, maybe due to the low *M*_n_ values, the *T*_g_ of **PUT-b** and **PUT-c** is not obvious (Figure S5).^45^ Thermogravimetric analysis (TGA) established that the PUs have good thermal stability, with a decomposition temperature (*T*_d_ 5%) above 221 °C (Figure S6). By simply regulating the reaction temperature in the one-pot reactions, PUs based on different diol linkers, named **PUS-a/b/c**, **PUD-a/b/c**, and **PUT-a/b/c**, respectively, gave different colored emission under a 365 nm UV lamp (Figures 1 and S7).

**Figure 1:**
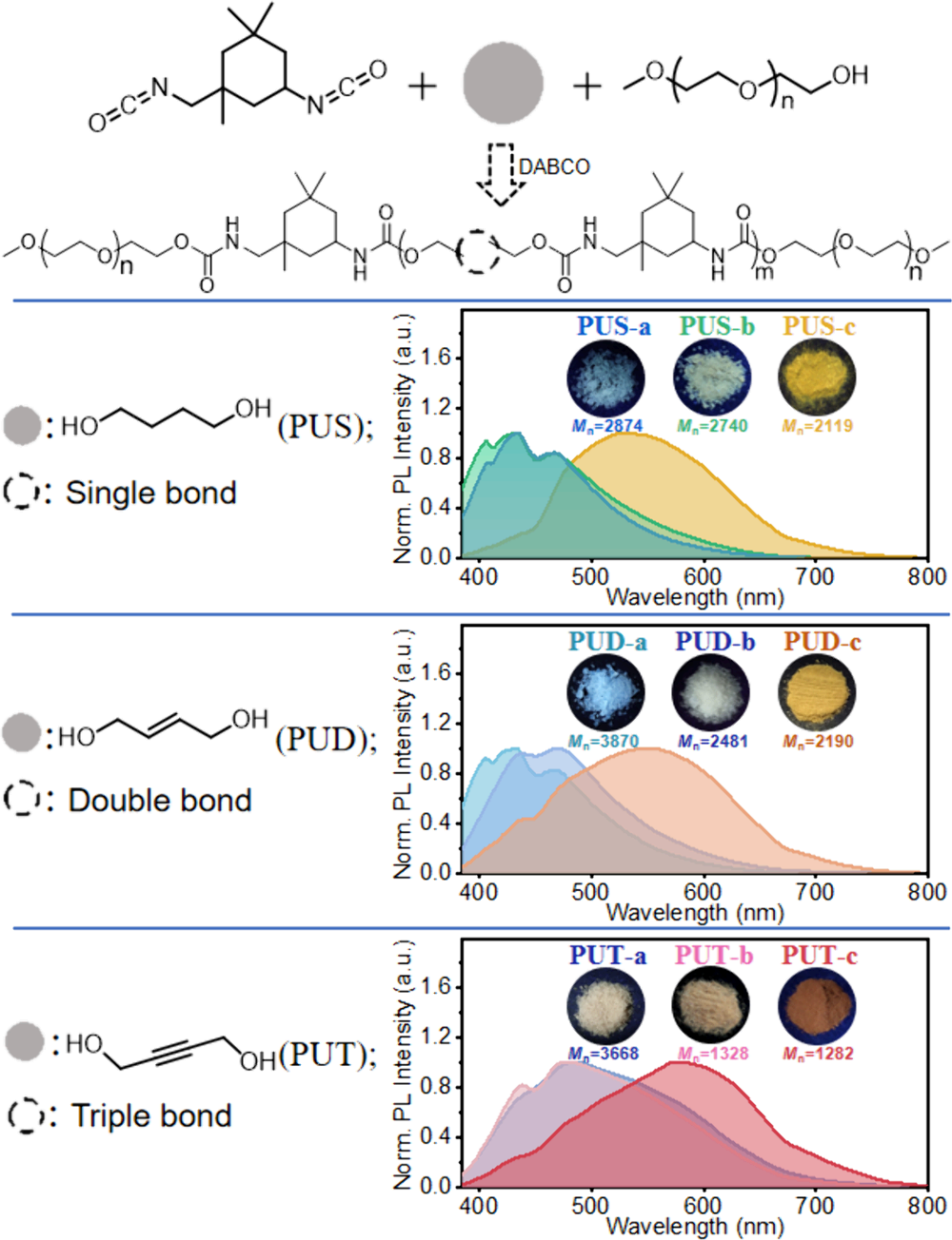
Synthetic route to **PUS**, **PUD**, and **PUT** (S, D, and T = single, double, and triple bond, respectively), the corresponding photoluminescence (PL) spectrum of powdered samples, number-average molecular weight (*M*_n_), and photographs under 365 nm UV illumination.

Within each series (**PUS-a/b/c**, **PUD-a/b/c**, and **PUT-a/b/c**) the polymers all have the same chemical structure; therefore, why are their luminescence properties different? First, the UV–visible absorption and excitation spectra before and after polymerization reactions (Figure S8) showed that the extent of polymerization influenced the width of the spectral profile. In Figure S9, the microscopic morphology of the PUs was monitored by scanning electron microscopy (SEM). Before polymerization, 1,4-butanediol, (*Z*)-2-butene-1,4-diol, and 2-butyne-1,4-diol monomers showed block/strip thin irregular morphologies. However, after polymerization, **PUS-a**, **PUD-a**, and **PUT-a** showed different degrees of microaggregation. **PUS-b/c**, **PUD-b/c**, and **PUT-b/c** showed similar micromorphological changes (Figure S10). Table S3 showed high-temperature products **PU-c** have higher molar absorbance coefficients than lower-temperature products **PU-a/b**,^46^ indicting their stronger intermolecular interactions and tighter packing.^47^ The extent of aggregation of the PUs positively correlated with their absorption and emission spectra. The longer wavelength emission of the lower *M*_n_ PUs is mainly due to the polymerization reaction whereby the rigid small molecular units become flexible polymer chains, segments of which are entangled, promoting the electron cloud overlap of electron-rich heteroatoms, resulting in strong through-space interactions.^9,22,48^ The fluorescence spectra of the PUs in the solid state showed that with the gradual increase of the excitation wavelength, the emission has a significant redshift and excitation-dependent characteristics (Figures S11–S13). The lifetime of the **PUS/D/T-c** products at different emission peaks and at different excitation wavelengths shows that the emission at different excitation wavelengths comes from different species (Figure S14).^13,17,19^ The absorption and emission behaviors of these samples at different concentrations in DMSO solvent were studied to assess their aggregate luminescence behavior. Figures 2 and S15–S16 show that all the PUs exhibit obvious concentration-enhanced emission characteristics.

**Figure 2:**
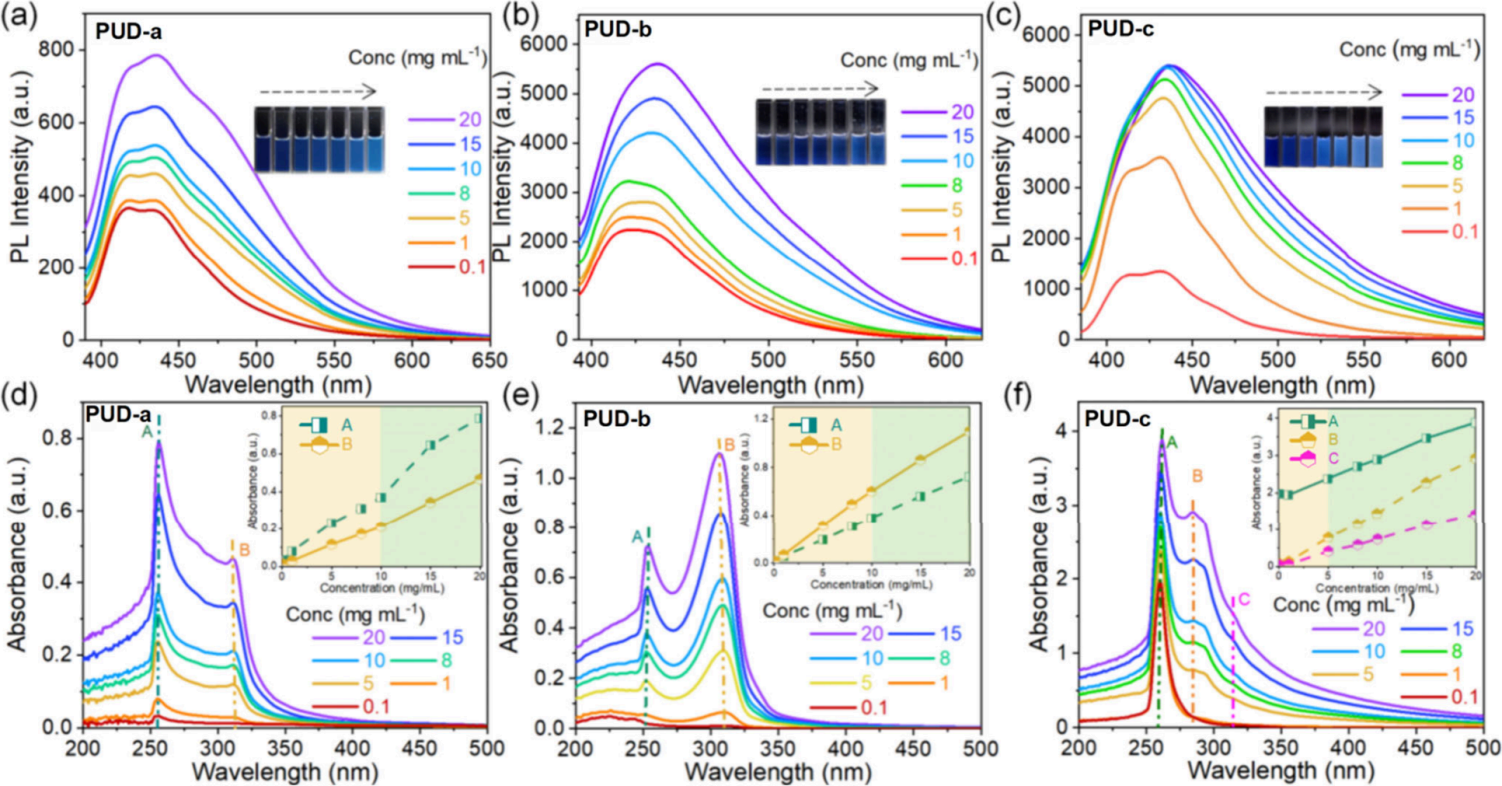
PL spectra of (a) **PUD-a**, (b) **PUD-b**, and (c) **PUD-c**; UV–vis spectra of (d) **PUD-a**, (e) **PUD-b**, and (f) **PUD-c** in DMSO solvent.

The B-peak of the PUs is stable at 300–310 nm (Figures 2 and S15–S16). In **PUS** and **PUT**, the position of peak B is stable. However, UV–vis of **PUD-c** has an extra peak C in place of the original peak B (Figure 2f). Thus, is it possible that peak C is really peak B? Peak B corresponds to S_1_, peak A corresponds to S_2_, and other minor peaks, such as peak C in Figure 2f and peak C/D in Figure S16e, may be the acromial peak generated by A/B vibration coupling. The absorption spectra of PUs demonstrate the complex and unpredictable aggregation of the polymer chains. Figure 2d–f shows the aggregation processes of the flexible chain segments at different concentrations and visualizes the existence of multiple emission species after aggregation. The specific content is discussed in the theory section below. Noteworthy, due to the more highly conjugated structure of **PUT**, the absorption of **PUT-c** solution exceeding 5 mg mL^–1^ is beyond the detection range of the instrument (Figure S16f); this also confirms that the spatial electron conjugation within the **PUT** series is much stronger than for **PUS** and **PUD**.

SEM was used to monitor the size and morphological changes of the microscopic aggregates over the concentration change. As shown in Figure 3, **PUS**, **PUD**, and **PUT** showed pronounced concentration-enhanced microscopic aggregation structures. At 0.5 mg mL^–1^ (Figure 3a,e,i), very sparse and small-sized nanostructures are observed. However, as the concentration increased, the PUs continued to aggregate, and at 10 mg mL^–1^ (Figure 3d,h,l) tightly clustered structures were observed. The accumulation of microscopic aggregates restricts molecular motion and nonradiative pathways, leading to a significant enhancement of fluorescence intensity (Figures 2, S15–S16). In addition, combining the emission characteristics of **PUS-c**, **PUD-c**, and **PUT-c**, it can be inferred that the cluster structure (Figure 3h,l) is more conducive to emission than the sheet structure (Figure 3d), which is reasonable, because the nonconjugated polymer mainly relies on through-space electron communication as the source of luminescence: at this level the cluster is more advantageous than the sheet structure.

**Figure 3:**
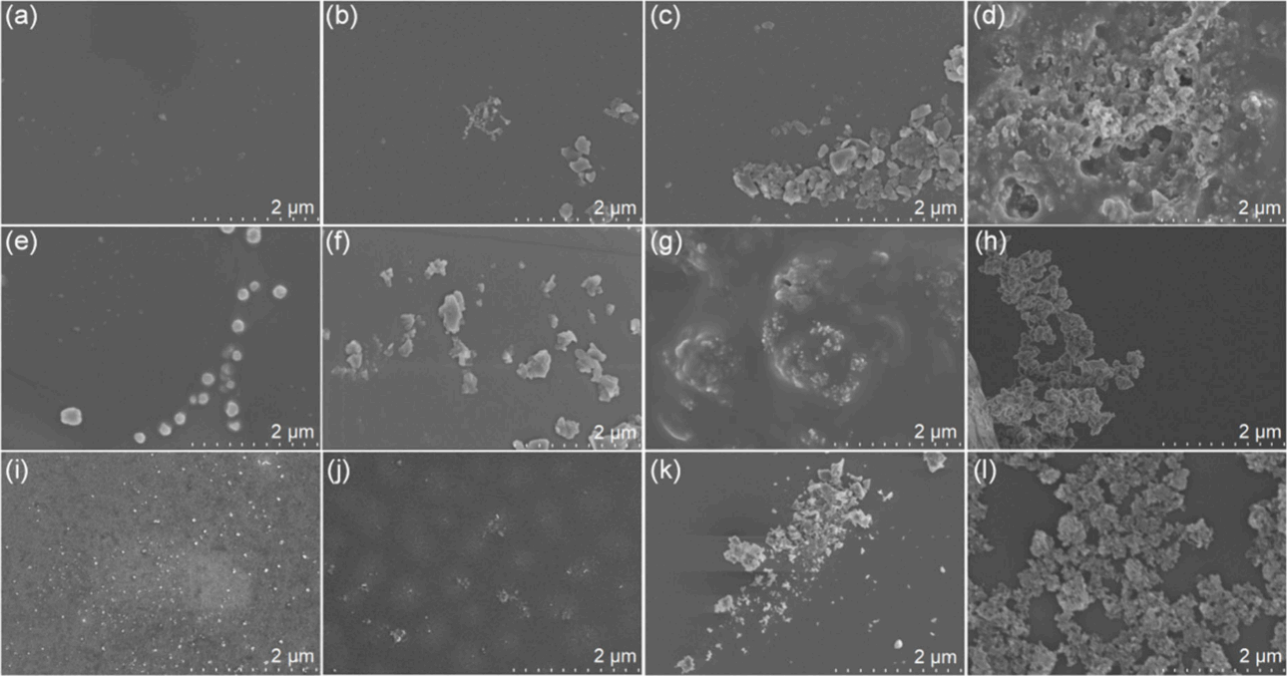
SEM images of **PUS-b** (a) 0.5 mg mL^–1^, (b) 1 mg mL^–1^, (c) 5 mg mL^–1^, and (d) 10 mg mL^–1^ in a DMSO solution. SEM images of **PUD-b** (e) 0.5 mg mL^–1^, (f) 1 mg mL^–1^, (g) 5 mg mL^–1^, and (h) 10 mg mL^–1^ in DMSO solution. SEM images of **PUT-b** (i) 0.5 mg mL^–1^, (j) 1 mg mL^–1^, (k) 5 mg mL^–1^, and (l) 10 mg mL^–1^ in DMSO solution.

Density functional theory (DFT) was used to calculate the lowest unoccupied and highest occupied molecular orbitals (LUMOs and HOMOs) based on two repeating units. As shown in Figure S17, the *E*_gap_ of **PUS**, **PUD**, and **PUT** decreases successively, consistent with their experimentally observed emission spectra. From the HOMO–LUMO perspective, **PUD** and **PUT** are similar; however, their luminous behaviors are quite different. Figure S18 shows that the triple-bonded **PUT** system has richer and stronger interchain/intrachain interactions, which may result in red-shifted emission. However, the exact source of multicolor emission and the detailed process of how weak interactions affect nonconjugated chromophores’ luminescence remain unclear (Table S4).

To elucidate the difference in photochemical properties between **PUS** and **PUD**/**PUT** with additional unsaturated moieties, two model systems, namely, **PUD-0** (ethylene + acetone) and **PUT-0** (acetylene + acetone), were constructed to exclude any influence of the polymer matrix environment. Smooth scan shows that excited state levels are highly related to the n-π interaction distance, i.e., the distance between the midpoints of two π bonds (Figure 4a). The lowest local excitation S_1_ state (n_1_ → π_1_*, Figure 4b) within C=O changes in parallel with the ground state, indicating that through-space n−π interaction does not influence the n_1_ → π_1_* transition. This corresponds well with the experimental UV–vis spectra, where a stable range of peak B (300–310 nm) is observed in all systems, regardless of the synthetic temperature or the surrounding π* acceptor (Figures 2, S15–S16). The levels of the S_2_ and S_3_ states decrease significantly with a smaller interaction distance, showing different behavior compared with other excited states. Natural transition orbitals (NTOs, Figure 4b) show that S_2_ and S_3_ have a strong through-space charge transfer (TSCT) feature from C=O to C=C, where a lone-pair orbital of an O atom (n_1_) or a π orbital of C=O (π_1_) acts as a donor in the electronic transition, while a π* orbital of C=C (π_2_*) acts as an acceptor. Considering that C=O and C=C bonds remain perpendicular with each other along the configuration scan, such through-space interactions should be featured as electron donations from n_1_ to π_2_*, as shown by the orbital interaction scheme in Figure 4c, with the most efficient overlap when the interaction angle is 90°.

**Figure 4:**
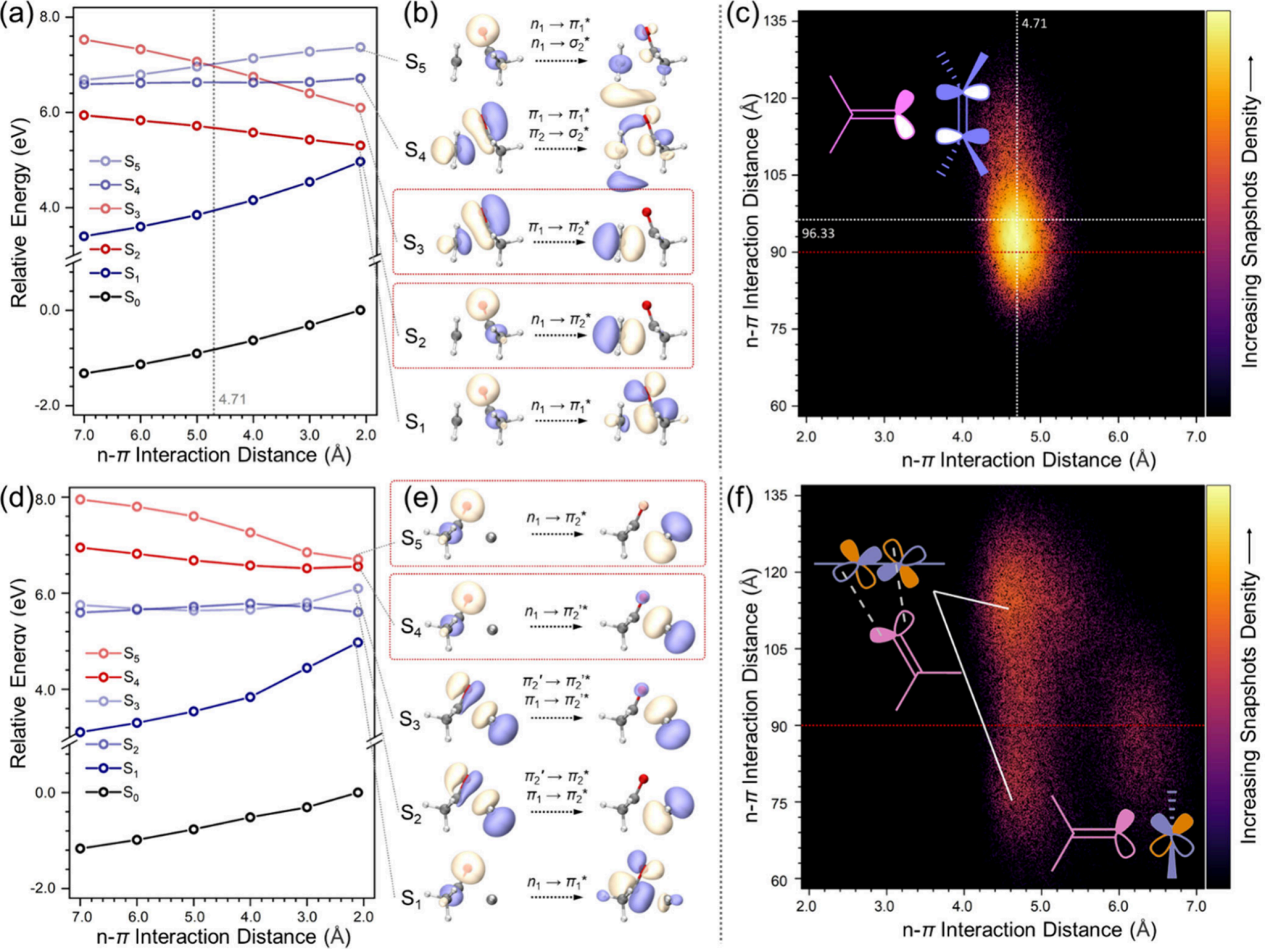
Excited-state level shift in C=O/C=C n-π interaction model system (a) **PUD-0**, (d) **PUT-0**. Excitation feature of lowest five excited states for (b) **PUD**, (e) **PUT**. Subscripts “1” and “2” denote C=O and C=C moieties, respectively. Density plot of n-π interaction distance and angle in MD3 trajectory of (c) **PUD-c**, (f) **PUT-c**.

Further MD simulations investigated noncovalent interactions in the aggregated environment. Each system involves a three-stage MD simulation, including (1) MD1: 50.0 ns equilibrium at 298 K, (2) MD2: 1.0 ns heating to synthetic temperature, then followed by 50.0 ns equilibrium, and (3) MD3: 1.0 ns annealing to 298 K, then followed by 50.0 ns equilibrium. As is shown in Figure 4d by density plot, statistics toward MD3 trajectory of **PUD-c** centers within a specific region, with an average interaction angle of 96.33°, being close to the ideal value of 90°. Since no special treatment was applied for the description of the lone-pair electrons and π* orbitals in the force field for the simulation, such results demonstrate that the through-space n−π interaction is favored by both the intrinsic electric field and the aggregated conformation in **PUD**. A similar conclusion also holds for **PUT** (Table S5). The interaction angle for **PUT** is retained as 96.35°, yet the average through-space n−π interaction distance is increased to 6.88 Å, compared with 4.71 Å in **PUD**. The electron density around C≡C is higher compared with C=C; therefore, through-space electron-donation from n_1_ to π_2_* is weakened, resulting in a larger interaction distance. In previous work, we have investigated the influence of hydrogen bonding in the aggregated system of nontraditional chromophores.^49−52^ By the formation of a hydrogen bond, the energy level of an *n* orbital increases to give a lower excitation energy, which should also be considered in **PUS/D/T** systems. Statistical results suggested that hydrogen bond networks in PU systems generally influence the excitation energy by −1.43 eV.^50^ According to Figure 4b, at the statistical distance (4.71 Å), the excitation energy of the S_2_ state in **PUD-0** is about 6.4 eV. Taking into account the influence of hydrogen bonds, the excitation energy is expected to be 4.97 eV (250 nm), which corresponds well to the experimental value of the A peak observed in UV–vis spectra (Figure 2) of **PUD-a/b/c** which originates from n_1_ → π_2_* charge transfer excitation, and both through-space n−π interactions and hydrogen bonds contribute to the color shift. Statistical results of hydrogen bonding and n−π interactions during the MD simulations for **PUS/D/T** show different temperature-dependent behaviors. For hydrogen bonds, a higher synthetic temperature leads to more aggregated structures as discussed,^49−52^ corresponding to an increase in the statistical number from the MD simulations (e.g., 159.8/143.7/173.3 for **PUS-a/b/c**, respectively). Different from hydrogen bonds, through-space n-π interactions show the opposite trend. The same statistical analysis was performed for the C–C bond at the same position of **PUS/D/T**. For **PUS**, the statistical number reveals a C–H/C=O interaction at a specific position, which is determined mainly by the electrostatic term and therefore shows a similar trend with hydrogen bonds. However, for **PUT**, the statistical number involves only through-space n-π interaction, showing nonlinear dependence on temperature. In the lower range of synthetic temperatures (**PUT-a/b**), through-space n-π interaction also contributes to more aggregated structures and thus has a similar trend with hydrogen bonds, while as revealed in Figure 4b, a more stable charge transfer excited state is accompanied by an increase in the ground-state energy, making through-space n-π interaction disfavored at higher temperature. This is demonstrated by MD simulations at 423 K, where the counts of through-space n-π interactions decrease sharply from 585.8 (**PUT-b**) to 510.1 (**PUT-c**). These results explain the abnormal photoluminescence (PL) intensity observed in **PUT-c**. Moreover, for **PUD** with both C–H/C=O and through-space n-π interaction taken into account, opposite temperature-dependent trends result in similar counts of interactions, indicating that **PUD** should be less sensitive to temperature.

**PUT** shares similar excited energy trends with **PUD**, but with the following differences. **PUT** has more low-lying excited states since C≡C has two π* orbitals acting as acceptor. Within the lowest five excited states investigated, there are two targeted n → π* transitions (S_4_ and S_5_, Figure 4d-f). Although with a similar transition feature, S_4_ involves electron transition on two perpendicular orbitals, being slightly lower than S_5_. S_2_ and S_3_ states show similar mixed features with mainly local excitation and minor charge transfer. The relative energy levels of S_2_ and S_3_ share similar energy splitting with the couple of S_4_ and S_5_. The n-π interaction in **PUT** is divided into two major configurations. With shorter n-π interaction distance (4.0–5.0 Å), the interaction angle distributes within a wide range of 70° ∼ 130°, especially two centers can be observed at ∼120° and ∼75°. Configurations with shorter interaction distance are joined by sloped n-π* orbital overlap, where two n orbitals of the oxygen atom interact with two carbon atoms (Figure 4f) to ensure efficient overlap. Meanwhile, configurations with longer interaction distance enable n orbitals to interact with π* at the same carbon atoms, therefore forming a perpendicular interaction angle (centered at ∼90°) and is much weaker than the previous sloped configuration. This can be seen from Figure 4f, where the long-distance configuration shows an obviously lower density. This also explains why **PUT-c** showed a much lower PL intensity compared with **PUT-a/b**. With higher temperature, short-distance configuration can be converted into long-distance configuration, while the latter is too weak to maintain a good aggregated structure.

The PUs exhibit different colors under ambient light or under a 365 nm UV lamp. **PUD-a**, **PUD-b**, **PUS-c**, and **PUT-c** were selected as assembly modules to verify the application potential of PUs in information transmission and colorful displays (Figure 5a). As shown in Figure S18, **PUD-a**, **PUD-b**, **PUS-c**, and **PUT-c** were used for pixel painting of a dog with a flower scarf. Under ambient light, this painting belongs to the dark series (Figure 5c). However, when illuminated by a 365 nm UV lamp, a bright dog is obtained with a light-blue luminous background, yellowish body, and blue-yellow scarf. **PUD-a** and **PUD-b** can also be used to construct a two-dimensional secret information display device (Figures S20–S21). Under daylight, the QR code did not scan any information (Figure S20a). However, under the irradiation of a 365 nm UV lamp, the information is revealed, and the information on “Macromolecules” can be swept out immediately (Figure S20b).

**Figure 5:**
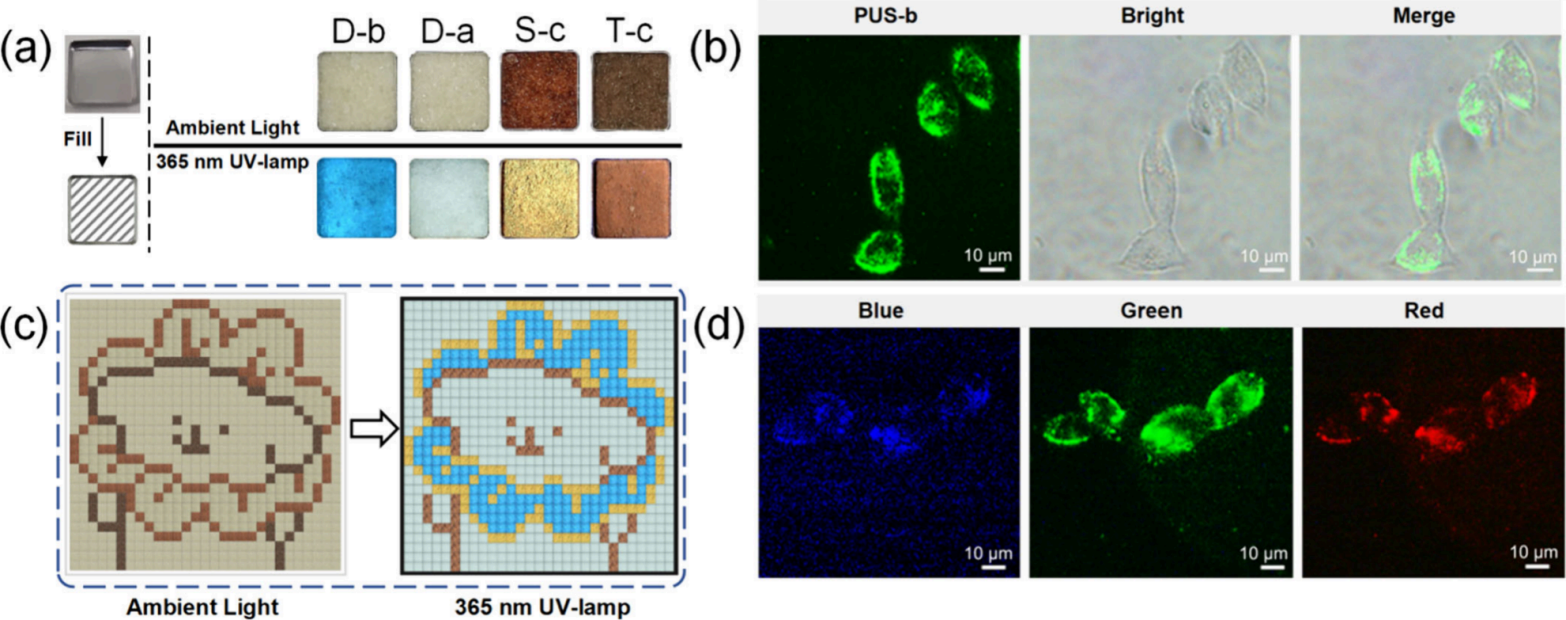
(a) Photographs of **PUD-b**, **PUD-a**, **PUS-c**, and **PUT-c** in daylight and ultraviolet light. (b) CLSM images of 4T1 cells incubated with 10 μg mL^–1^**PUS-b** for 6 h under dark field, bright field, and merged images. (c) The pixel painting and its photo in ambient light and 365 nm UV-lamp, respectively. (d) CLSM images of 4T1 cells incubated with 10 μg mL^–1^**PUT-c** for 6 h under blue, green, and red channels in the cells.

In addition, due to the significant fluorescence and excitation-dependent emission characteristics of the PUs, their potential application in multifunctional bioimaging was explored. Taking **PUS-b** and **PUT-c** as examples, as shown in Figure 5b, after being incubated with 4T1 cells for 6 h, **PUS-b** successfully penetrated the cell membrane, and the cells showed bright green fluorescence, representing an efficient fluorescent probe. 4T1 cells were incubated with 10 μg mL^–1^**PUT-c** (with both the longest wavelength emission and the strongest excitation-dependent property among the PUs) for 6 h, and then the cells were captured by confocal laser scanning microscopy (CLSM) under three channels. Figure 5d showed bright fluorescence in the 4T1 cells under blue, green, and red channels. MTT assay showed that **PUS-b** and **PUT-c** have negligible cytotoxicity toward 4T1 cells (Figure S22), demonstrating their biological safety. Multichannel fluorescence imaging is a valuable tool for better studying biological processes,^53^ and PUs can, therefore, be considered as a promising multifunctional fluorescence probe.

In summary, this comprehensive study of polyurethane (PU) derivatives with single, double, or triple bond repeating units in the main chain has identified how the different effects of H-bonds and through-space n-π interactions achieve full-color luminescence (red, green, and blue). The detailed photophysical characterization and theoretical calculations reveal that the excited-state levels are highly related to the n-π interaction distances. The short-wavelength absorption observed in **PUD-a/b/c** originates from n_1_ → π_2_* charge transfer excitation, and both through-space n-π interactions and hydrogen bonds contribute to the long-wavelength absorption. Such through-space n-π interactions are favored by both the intrinsic electric field and the aggregated conformation in **PUD**. Statistical results of hydrogen bonding and n-π interactions during the MD simulations for **PUS/D/T** show different temperature-dependent behaviors. For hydrogen bonds, a higher synthetic temperature leads to better aggregated structures. Through-space n-π interactions show a similar trend in a lower range of synthetic temperature; however, higher temperature disfavored stable through-space n-π interactions, resulting in a decrease in PL intensity and quantum yield. The potential applications of the PUs in colorful displays, covert information transmission, and multifunctional bioimaging have been verified. This work provides a new protocol for the simple preparation of multifunctional fluorescent polymers, while also deepening the understanding of the mechanism of luminescence in the aggregated state of nonconventional luminophores.

## Data Availability

The data associated with this article is available in the manuscript and Supporting Information files.

## Supporting Information Available

Experimental section; structure analysis; additional photophysical data; scanning electron microscopy (SEM) data; computational data (PDF)
