# Supplementary material for: Nonconventional Full-Color Luminescent Polyurethanes: Luminescence Mechanism at the Molecular Orbital Level
Source: ACS Mater Lett. 2024 Nov 21;7(1):24–31. doi: 10.1021/acsmaterialslett.4c02100 (PMC11707742; doi:10.1021/acsmaterialslett.4c02100)
Supplement: Supplementary file 1 — tz4c02100_si_001.pdf [file tz4c02100_si_001.pdf]

Supporting Information

Nonconventional Full-color Luminescent Polyurethanes: Luminescence Mechanism at the Molecular Orbital Level

Nan Jiang,[a] Ya-Jie Meng,[b] Xin Pu,[c] Chang-Yi Zhu,[a] Shu-Han Tan,[a] Yan-Hong Xu,[*a] You-Liang Zhu,[*c] Jia-Wei Xu,[b] Martin R. Bryce[*d]

[a] Key Laboratory of Preparation and Applications of Environmental Friendly Materials, Key Laboratory of Functional Materials Physics and Chemistry of the Ministry of Education (Jilin Normal University), Changchun, 130103, China.
[b] Ministry-of-Education Key Laboratory of Numerical Simulation of Large-Scale Complex System (NSLSCS) and School of Chemistry and Materials Science, Nanjing Normal University, Nanjing 210023, China.
[c] State Key Laboratory of Supramolecular Structure and Materials, College of Chemistry, Jilin University, Changchun 130012, China.
[d] Department of Chemistry, Durham University, Durham DH1 3LE, U.K.
E-mails: xuyh198@163.com; youliangzhu@jlu.edu.cn; m.r.bryce@durham.ac.uk

Contents:

1. Experimental details

2. Structural characterization

3. Photophysical properties

4. Theoretical calculations

5. Applications

6. References

1. Experimental details

Materials

Materials obtained from commercial suppliers were used without further purification unless otherwise stated. All glassware, syringes, magnetic stirring bars, and needles were thoroughly dried in a convection oven.

Characterization

The UV-vis absorption spectra were recorded on a Shimadzu UV-3100 spectrophotometer. The luminescence movie and photos were taken by an iPhone 14 pro under the irradiation of a hand-held UV lamp at room temperature. ^{1}H NMR spectra were recorded on a Varian 500 MHz spectrometer. The ^{1}H NMR spectra were referenced internally to the residual proton resonance in DMSO-d_6 (δ 2.5 ppm). The molecular weights of the polyurethane were determined by gel permeation chromatography (GPC) on a Waters 410 instrument with monodispersed polystyrene as the reference and THF as the eluent at 35°C. Scanning electron microscope (SEM) images were obtained using a JEOL model JSM-6700 instrument operating at an accelerating voltage of 5.0 kV. The samples were prepared by placing microdrops of the solution on a holey carbon copper grid. Steady-state photoluminescence/phosphorescence spectra and phosphorescence lifetime were measured using a Hitachi F-4700 instrument. The fluorescence lifetime was obtained on an Edinburgh FLS-1000 instrument. The photoluminescence quantum efficiency was obtained on a Hitachi F-4700 instrument. DSC data were obtained using a NETZSCH thermal analysis DSC 214 instrument under argon with a heating rate of 10 °C min^{-1}. TGA measurements were performed on a Discovery TGA under N_2, by heating from 40 to 800 °C at a rate of 10 °C min^{-1}.

Synthesis of PUS-a

A mixture of 1,4-butanediol (0.236 g, 2.62 mmol), polyethylene glycol mono-methyl ether (M_w = 350 g mol^{-1}; 0.693 g, 1.98 mmol), anhydrous THF (5 mL), isophorone diisocyanate (0.802 g, 3.61 mmol) and 1,4-diazabicyclooctane triethylenediamine (DABCO) (0.012 g, 0.105 mmol) was stirred in N_2 atmosphere at 65°C for 8 h until the clear solution became viscous, indicating that polymerization had occurred. After cooling to room temperature, the mixture was added to excess *tert*-butyl methyl ether drop by drop for reverse precipitation to give a product which was then dried under vacuum at room temperature for 24 h to obtain polyurethane **PUS-a** (0.80 g, 46% yield).

Synthesis of PUS-b

A mixture of 1,4-butanediol (0.236 g, 2.62 mmol), polyethylene glycol mono-methyl ether (M_w = 350 g mol^{-1}; 0.693 g, 1.98 mmol), anhydrous THF (5 mL), isophorone diisocyanate (0.802 g, 3.61 mmol) and 1,4-diazabicyclooctane triethylenediamine (DABCO) (0.012 g, 0.105 mmol) was stirred in N_2 atmosphere at 75°C for 8 h until the clear solution became viscous, indicating that polymerization had occurred. After cooling to room temperature, the mixture was added to excess *tert*-butyl methyl ether drop by drop for reverse precipitation to give a

product which was then dried under vacuum at room temperature for 24 h to obtain polyurethane **PUS-b** (0.84 g, 48% yield).

Synthesis of PUS-c

A mixture of 1,4-butanediol (0.236 g, 2.62 mmol), polyethylene glycol mono-methyl ether (M_w = 350 g mol^{-1}; 0.693 g, 1.98 mmol), anhydrous DMSO (5 mL), isophorone diisocyanate (0.802 g, 3.61 mmol) and 1,4-diazabicyclooctane triethylenediamine (DABCO) (0.012 g, 0.105 mmol) was stirred in N_2 atmosphere at 150°C for 24 h until the clear solution became viscous, indicating that polymerization had occurred. After cooling to room temperature, the mixture was added to excess *tert*-butyl methyl ether drop by drop for reverse precipitation to give a product which was then dried under vacuum at room temperature for 24 h to obtain polyurethane **PUS-c** (0.78 g, 45% yield).

Synthesis of PUD-a

A mixture of (*Z*)-2-butene-1,4-diol (0.231 g, 2.62 mmol), polyethylene glycol mono-methyl ether (M_w = 350 g mol^{-1}; 0.693 g, 1.98 mmol), anhydrous THF (5 mL), isophorone diisocyanate (0.802 g, 3.61 mmol) and 1,4-diazabicyclooctane triethylenediamine (DABCO) (0.012 g, 0.105 mmol) was stirred in N_2 atmosphere at 65°C for 8 h until the clear solution became viscous, indicating that polymerization had occurred. After cooling to room temperature, the mixture was added to excess *tert*-butyl methyl ether drop by drop for reverse precipitation to give a product which was then dried under vacuum at room temperature for 24 h to obtain polyurethane **PUD-a** (0.84 g, 49% yield).

Synthesis of PUD-b

A mixture of (*Z*)-2-butene-1,4-diol (0.231 g, 2.62 mmol), polyethylene glycol mono-methyl ether (M_w = 350 g mol^{-1}; 0.693 g, 1.98 mmol), anhydrous THF (5 mL), isophorone diisocyanate (0.802 g, 3.61 mmol) and 1,4-diazabicyclooctane triethylenediamine (DABCO) (0.012 g, 0.105 mmol) was stirred in N_2 atmosphere at 75°C for 8 h until the clear solution became viscous, indicating that polymerization had occurred. After cooling to room temperature, the mixture was added to excess *tert*-butyl methyl ether drop by drop for reverse precipitation to give a product which was then dried under vacuum at room temperature for 24 h to obtain polyurethane **PUD-b** (0.94 g, 55% yield).

Synthesis of PUD-c

A mixture of (*Z*)-2-butene-1,4-diol (0.231 g, 2.62 mmol), polyethylene glycol mono-methyl ether (M_w = 350 g mol^{-1}; 0.693 g, 1.98 mmol), anhydrous DMSO (5 mL), isophorone diisocyanate (0.802 g, 3.61 mmol) and 1,4-diazabicyclooctane triethylenediamine (DABCO) (0.012 g, 0.105 mmol)was stirred in N_2 atmosphere at 150°C for 24 h until the clear solution became viscous, indicating that polymerization had occurred. After cooling to room temperature, the mixture was added to excess *tert*-butyl methyl ether drop by drop for reverse precipitation to give a product which was then dried under vacuum at room temperature for 24 h to obtain polyurethane **PUD-c** (0.89 g, 51% yield).

Synthesis of PUT-a

A mixture of 2-butyne-1,4-diol (0.226 g, 2.62 mmol), polyethylene glycol mono-methyl ether (M_w = 350 g mol^{-1}; 0.693 g, 1.98 mmol), anhydrous THF (5 mL), isophorone diisocyanate (0.802 g, 3.61 mmol) and 1,4-diazabicyclooctane triethylenediamine (DABCO) (0.012 g, 0.105 mmol) was stirred in N_2 atmosphere at 65°C for 8 h until the clear solution became viscous, indicating that polymerization had occurred. After cooling to room temperature, the mixture was added to excess *tert*-butyl methyl ether drop by drop for reverse precipitation to give a product which was then dried under vacuum at room temperature for 24 h to obtain polyurethane **PUT-a** (0.97 g, 57% yield).

Synthesis of PUT-b

A mixture of 2-butyne-1,4-diol (0.226 g, 2.62 mmol), polyethylene glycol mono-methyl ether (M_w = 350 g mol^{-1}; 0.693 g, 1.98 mmol), anhydrous THF (5 mL), isophorone diisocyanate (0.802 g, 3.61 mmol) and 1,4-diazabicyclooctane triethylenediamine (DABCO) (0.012 g, 0.105 mmol) was stirred in N_2 atmosphere at 75°C for 8 h until the clear solution became viscous, indicating that polymerization had occurred. After cooling to room temperature, the mixture was added to excess *tert*-butyl methyl ether drop by drop for reverse precipitation to give a product which was then dried under vacuum at room temperature for 24 h to obtain polyurethane **PUT-b** (1.05 g, 61% yield).

Synthesis of PUT-c

A mixture of 2-butyne-1,4-diol (0.226 g, 2.62 mmol), polyethylene glycol mono-methyl ether (M_w = 350 g mol^{-1}; 0.693 g, 1.98 mmol), anhydrous DMSO (5 mL), isophorone diisocyanate (0.802 g, 3.61 mmol) and 1,4-diazabicyclooctane triethylenediamine (DABCO) (0.012 g, 0.105 mmol) was stirred in N_2 atmosphere at 150°C for 24 h until the clear solution became viscous, indicating that polymerization had occurred. After cooling to room temperature, the mixture was added to excess *tert*-butyl methyl ether drop by drop for reverse precipitation to give a product which was then dried under vacuum at room temperature for 24 h to obtain polyurethane **PUT-c** (1.07 g, 62% yield).

Molar Absorption Coefficient

The molar absorption coefficient of PUs, $\varepsilon(\mathrm{PUs})_{\lambda\mathrm{abs}}$, L·mol^{-1}·cm^{-1}, was calculated by the equation of the Bouguer-Lambert-Beer law

$$\varepsilon(\mathrm{PUs})_{\lambda\mathrm{abs}} = A(\mathrm{PUs})_{\lambda\mathrm{abs}} /([\mathrm{PUs}] \times L),$$

where $A(\mathrm{PUs})_{\lambda\mathrm{abs}}$ is the true absorbance of PUs in solution at the absorption maximum λ_{abs} nm; [PUs] is the concentration of PUs in solution, mol/L; and L = 1 cm is the optical path of spectrophotometer cells.[S1]

Density functional theory (DFT) calculations

Density functional theory (DFT) calculations were all performed using the Gaussian 16 C.01 program[S2] at the B3LYP/6-31G(d) level. The DFT calculations were performed on the polyurethane with two repeating units.

Molecular Dynamics

Three molecular dynamics systems for PUS, PUD and PUT (m = 2 and n = 2) were built by Packmol program. Corresponding isolated molecular models was optimized at the PBE0-D3(BJ)/def2-SVP level of theory [S2,S3] using Gaussian 16 (Revision C.01) [S4] and no imaginary frequency was checked by frequency calculation. The restrained electrostatic potential (RESP) atomic charges were generated by Multiwfn. [S5] Force field parameters were adopted from generalized Amber force field (GAFF). [S6]

Molecular dynamics (MD) simulations were performed using the GROMACS (version 2022.5) package [S7] and topology file and forcefield parameters were created by Sobtop. [S8] The long-range electrostatic interactions were handled by the particle-mesh Ewald (PME) method and the cutoff value of van der Waals interactions was set to 10.0 Å.[S9] After energy minimization, the three systems were heated up from 0 K to 300 K in the 1.0 ns simulations. Subsequently, the 50.0 ns MD simulations (MD1) were conducted in the NPT ensemble at 300 K using the v-rescale thermostat method[S10] and the Berendsen.[S11] Next, both systems were heated up to synthetic temperature (338 K, 348 L and 423 K for PUX-a, PUX-b and PUX-c, respectively), with the 50.0 ns of MD simulations (MD2). Finally, the temperature of the three systems dropped to 300 K and unrestrained MD simulations for 50.0 ns (MD3) were performed.

QM Calculation

To investigate to function of C=C and C≡C moieties, two model system, namely PUD-0 and PUT-0 were designed for QM calculation. Scan along the through-space π-π interaction direction was performed using Gaussian 16 program under the theory of M06-2X-D3/def2-TZVP by adding generalized internal coordinates (GIC). The GIC used from smooth scan was defined as the distance between midpoints of C=C/C≡C and C=O bonds, utilizing 0.10 Å step size for scanning to ensure no unexpected configuration change during the scan coordinates. The snapshots were taken every 10 scan steps (1.0 Å) for following multi-reference studies.

Natural transition orbitals (NTO) were calculated under NEVPT2/def2-TZVP level of theory. Active spaces used for multi-reference calculation was selected by standard workflow of MOKIT[S12] based on the optimized structure, including: 1). RHF/def2-TZVP single point calculation was performed with background charges and wavefunction was checked to be stable by Gaussian16; 2). CIS/def2-TZVP calculation with background charges was performed for lowest 7 states based on previous RHF wavefunction and all excitation components with contribution larger than 10^{-5} were considered and 3). Active spaces were determined based on NTO obtained in CIS calculation.[S13] NEVPT2/MM calculation was performed by PySCF package (version 2.5.0).[S14]

Radial distribution function

The calculation equation for radial distribution function (RDF) can be described as:[S15, S16]

$$g(r) = \lim_{dr \to 0} \frac{p(r)}{4\pi \left(\frac{N_{pairs}}{V} \right) r^2 dr}$$

where r is the distance between each atom pair, $g(r)$ is radial distribution function, $p(r)$ is the average number of atom pairs. N_{pairs} is the total number of atom pairs, V is the volume of the simulation cell.

Cell culture method

Mouse breast cancer cells (4T1 cells) were selected as the cell type for this experiment. First, Roswell Park Memorial Institute (RPMI) 1640 medium containing 10% fetal bovine serum by volume was configured, and the cell culture vial was placed in an incubator at a temperature of 37 ℃ and 5% CO_2 for culture. In order to ensure that the cells have sufficient nutrients, the medium was changed every two days.

Cell imaging

Confocal laser scanning microscopy (CLSM) was used for imaging of the material on the cells, and a 1 mL cell suspension was added to the confocal petri dish at a density of 50,000 cells per well. The cell culture vial was placed in an incubator overnight. The original medium was extracted, 1 mL of medium containing material (10 μg mL^{-1}) was added, and cultured in the incubator for 6 h. The cell imaging by CLSM used λ_{ex}= 380-410 nm, λ_{em}= 415-475 nm for the blue channel; λ_{ex}= 465-495 nm, λ_{em}= 515-555 nm for the green channel; λ_{ex}= 540-580 nm, λ_{em}= 600-660 nm for the red channel.

Cytotoxicity test method

The cytotoxicity of the materials was determined by 3-(4,5-dimethylthiazol-2-yl)-2,5-dipheny l-2*H*-tetrazolium bromide (MTT) assay. 4T1 cells were placed into 96-well plates at a density of 10,000 cells per well, that is, 100 μL cell suspension was added into each well. The 96-well plates were incubated overnight in an incubator for cell adhesion growth. The media were then removed, and then media containing different concentrations of materials (0-40 μg mL^{-1}) were added to the cell pore plates, each 100 μL. The cells were cultured in an incubator for 24 h. Then 10 μL of MTT (5 mg mL^{-1}) was added to each well and cultured in an incubator for 4 h. The medium was replaced with DMSO (200 μL). The absorbance at a reference wavelength of 490 nm was recorded on an enzyme-labeler.

2. Structural characterization

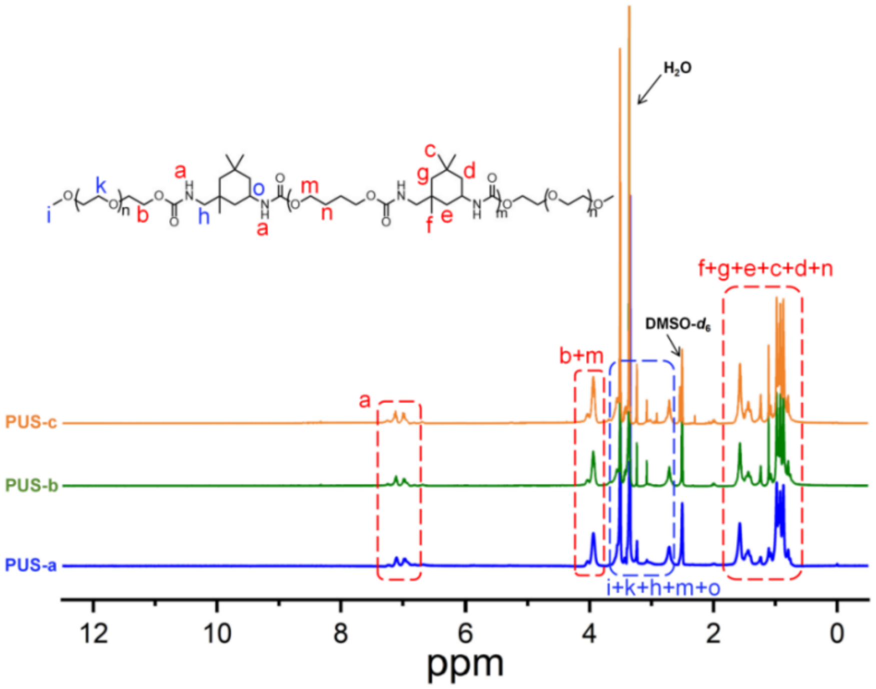

Figure S1. ^{1}H NMR spectra of **PUS** in DMSO-d_6.

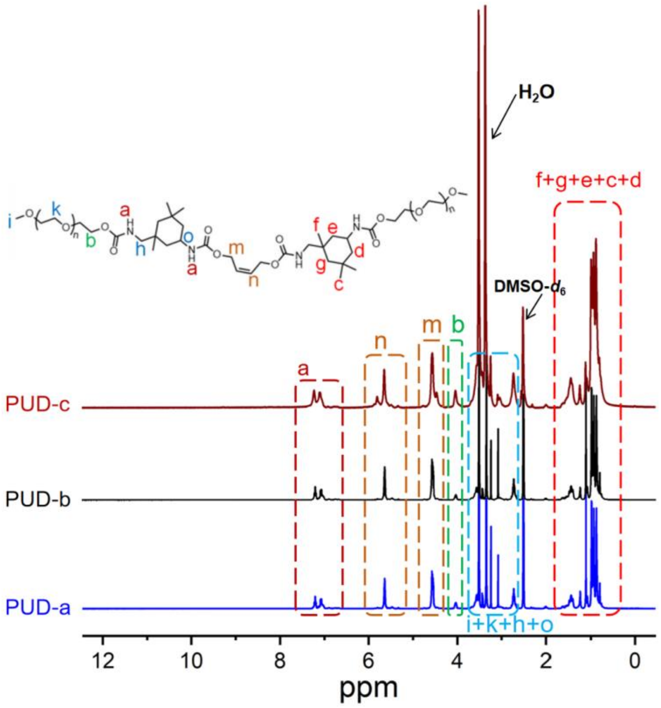

Figure S2. ^{1}H NMR spectra of **PUD** in DMSO-d_6.

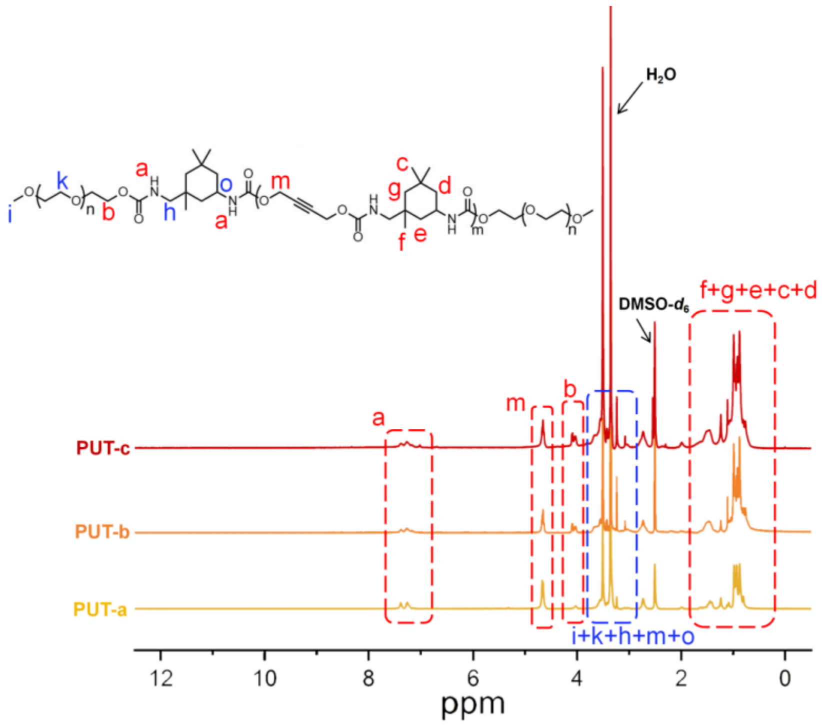

Figure S3. ^{1}H NMR spectra of **PUT** in DMSO-d_6.

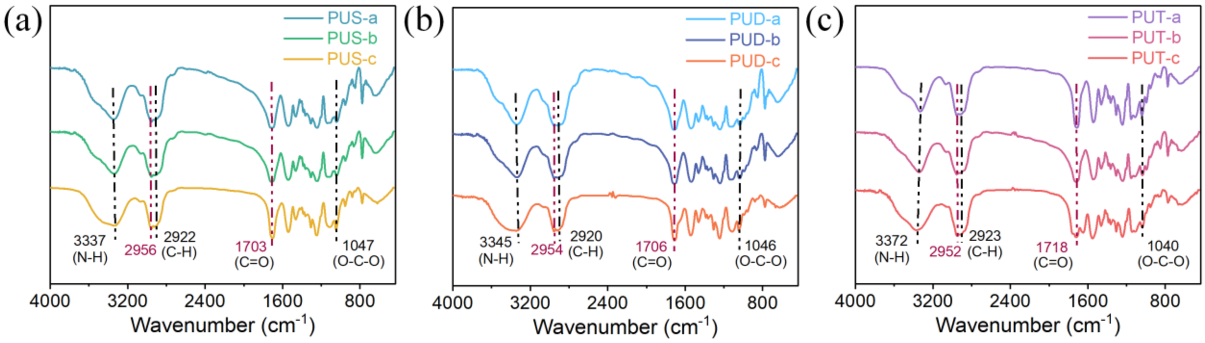

Figure S4. FT-IR spectra of **PUS/PUD/PUT** series powder samples.

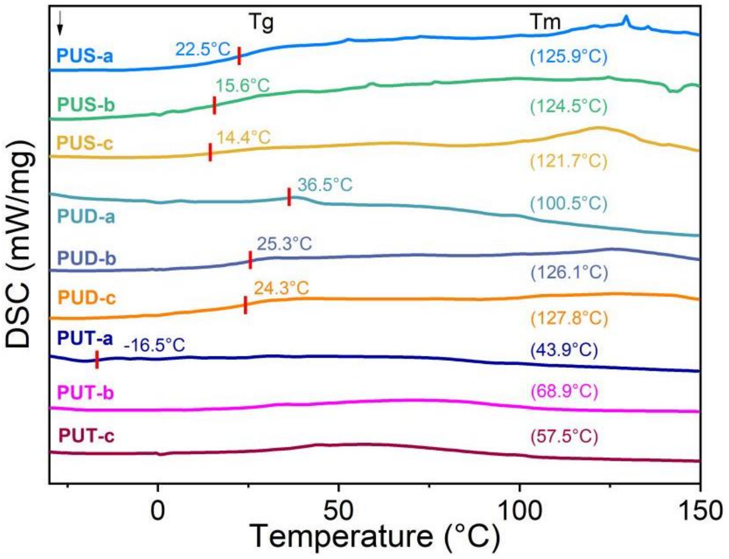

Figure S5. DSC curves of PUs powder samples.

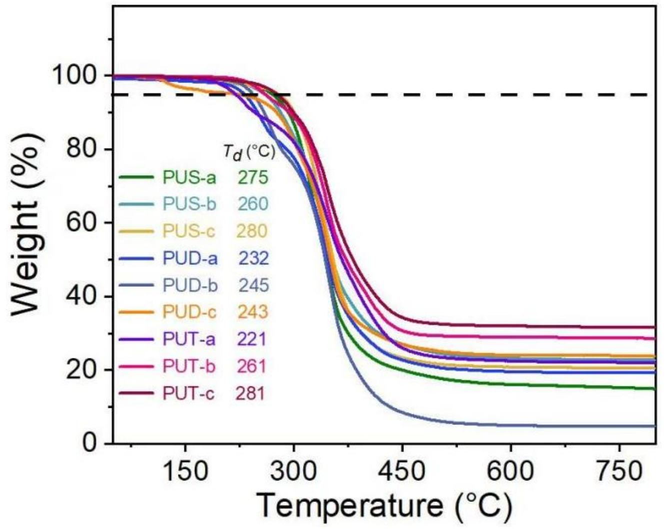

Figure S6. TGA curves of PUs.

3. Photophysical properties

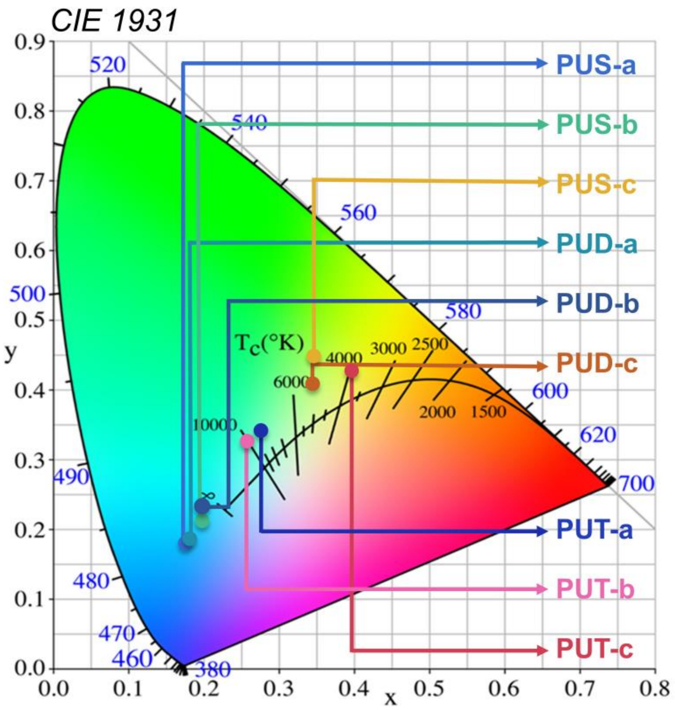

Figure S7. CIE chromaticity diagram of fluorescent emission of **PUS/PUD/PUT** powder samples at room temperature (λ_{ex} = 365 nm).

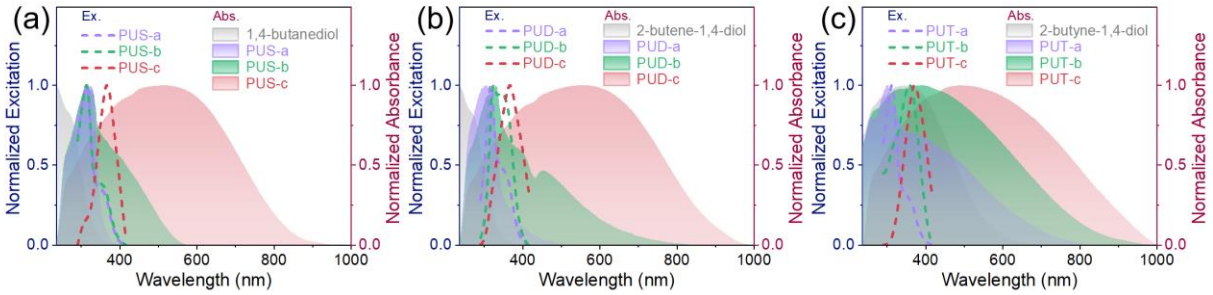

Figure S8. Normalized excitation and UV-vis absorption spectra of (a) **PUS**, (b) **PUD** and (c) **PUT** powder samples at room temperature.

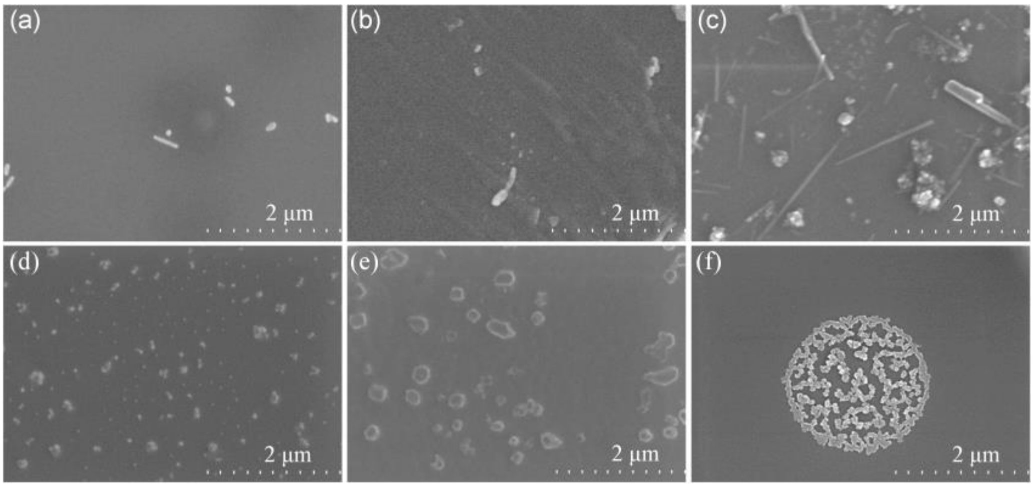

Figure S9. SEM images of 20 mg mL^{-1} (a) 1,4-butanediol, (b) (*Z*)-2-butene-1,4-diol, (c) 2-butyne-1,4-diol monomers (d) **PUS-a**, (e) **PUD-a**, (f) **PUT-a** products dispersed in ethanol.

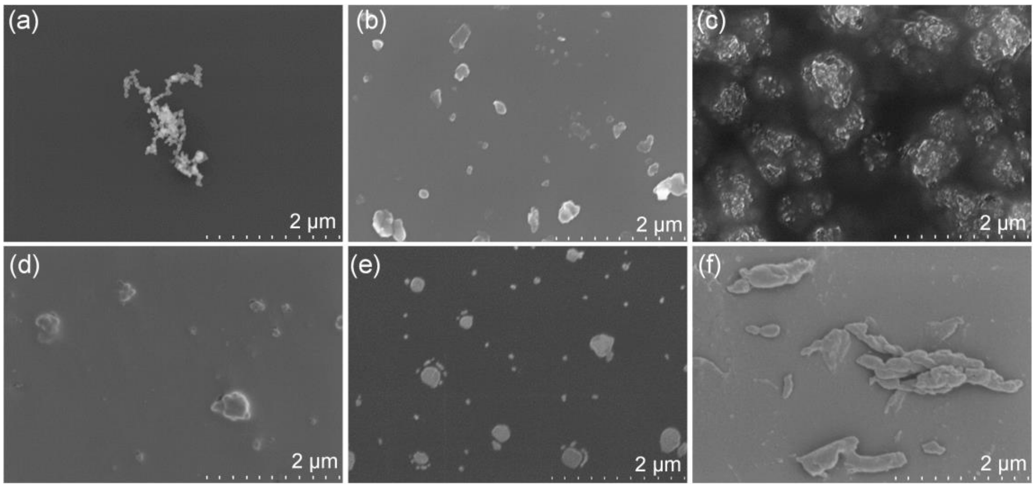

Figure S10. SEM images of (a) **PUS-b**, (b) **PUD-b**, (c) **PUT-b**, (d) **PUS-c**, (e) **PUD-c**, (f) **PUT-c** dispersed in ethanol (20 mg mL^{-1}).

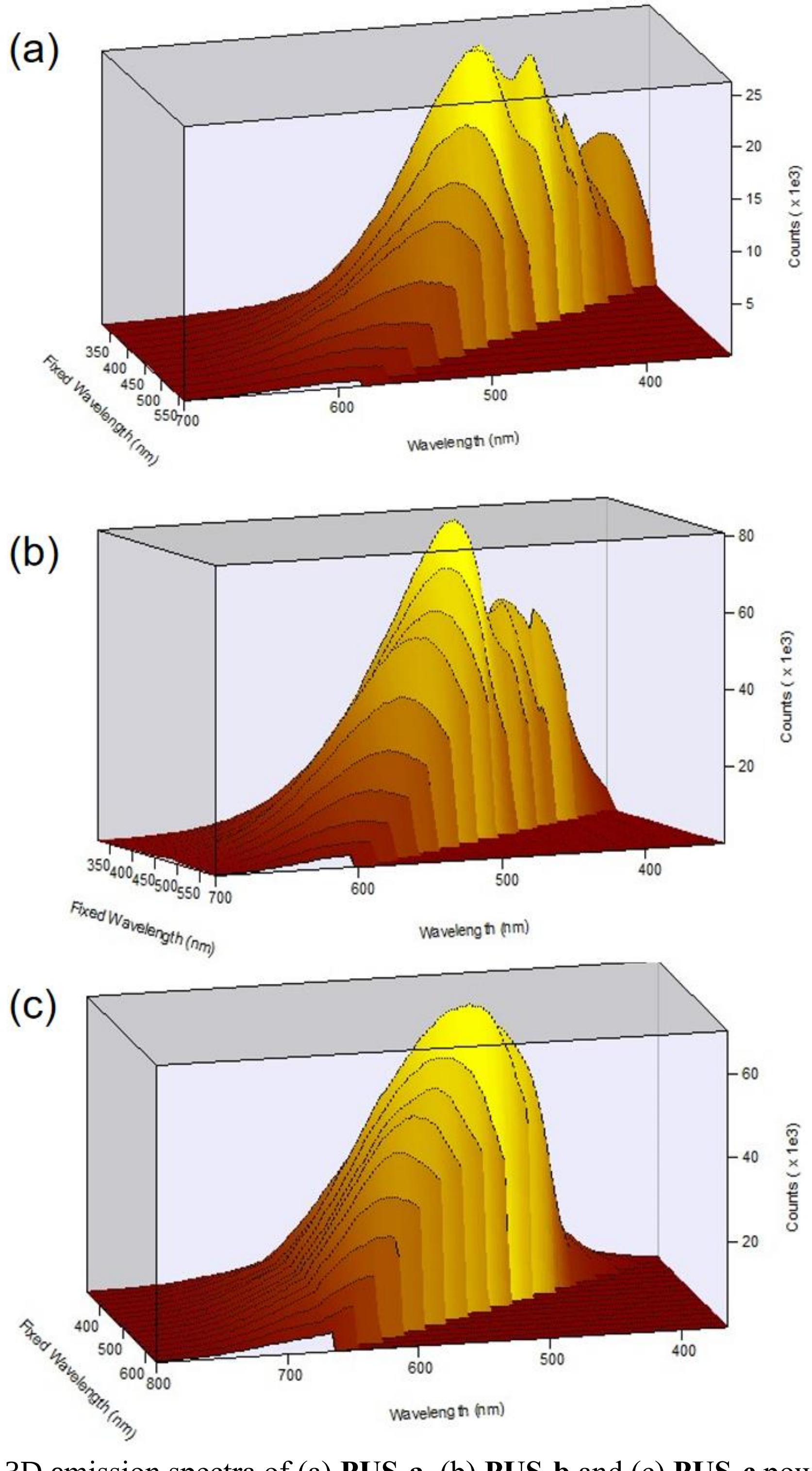

Figure S11. 3D emission spectra of (a) **PUS-a**, (b) **PUS-b** and (c) **PUS-c** powder samples with different excitation wavelengths at room temperature.

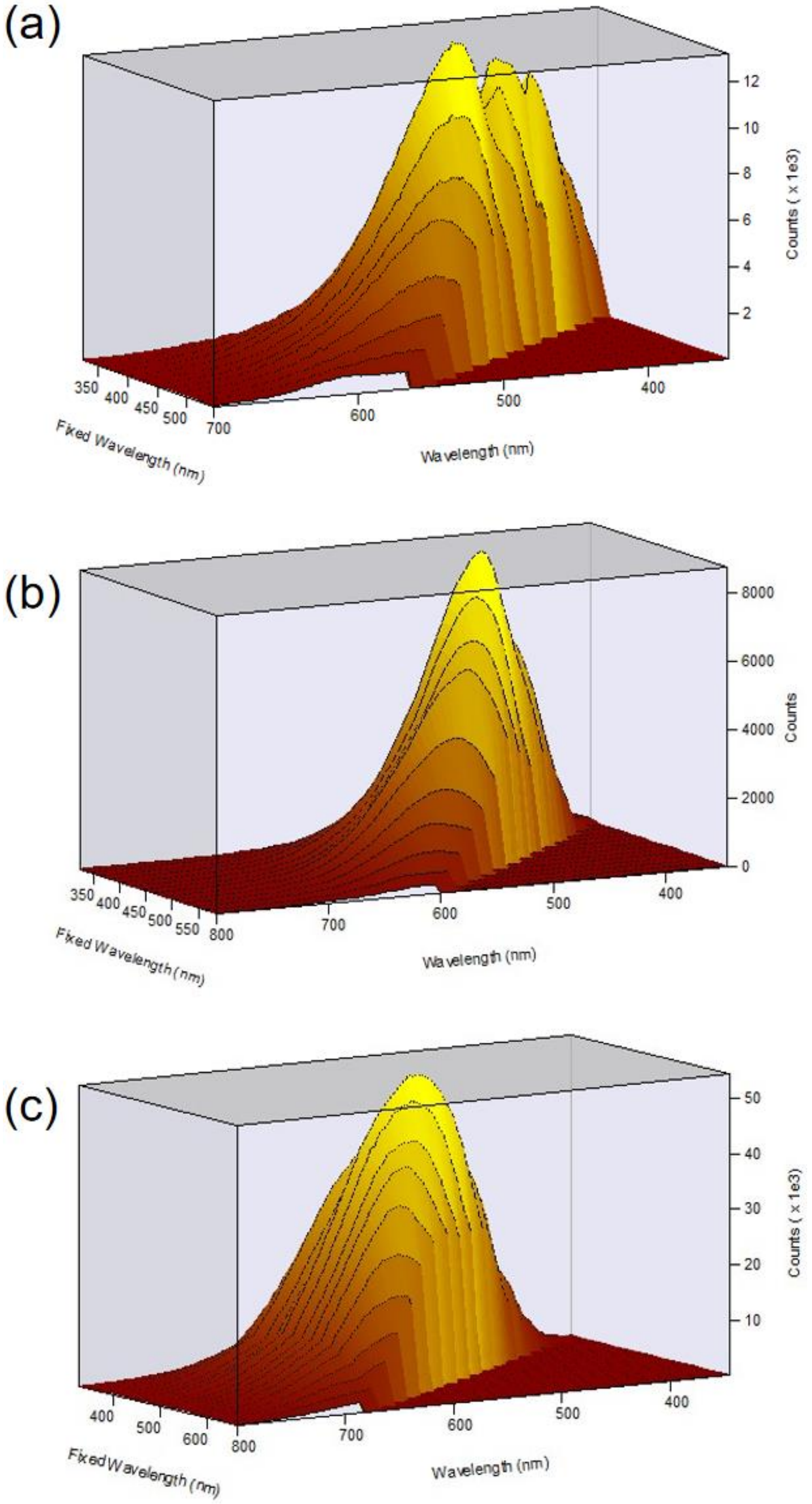

Figure S12. 3D emission spectra of (a) **PUD-a**, (b) **PUD-b** and (c) **PUD-c** powder samples with different excitation wavelengths at room temperature.

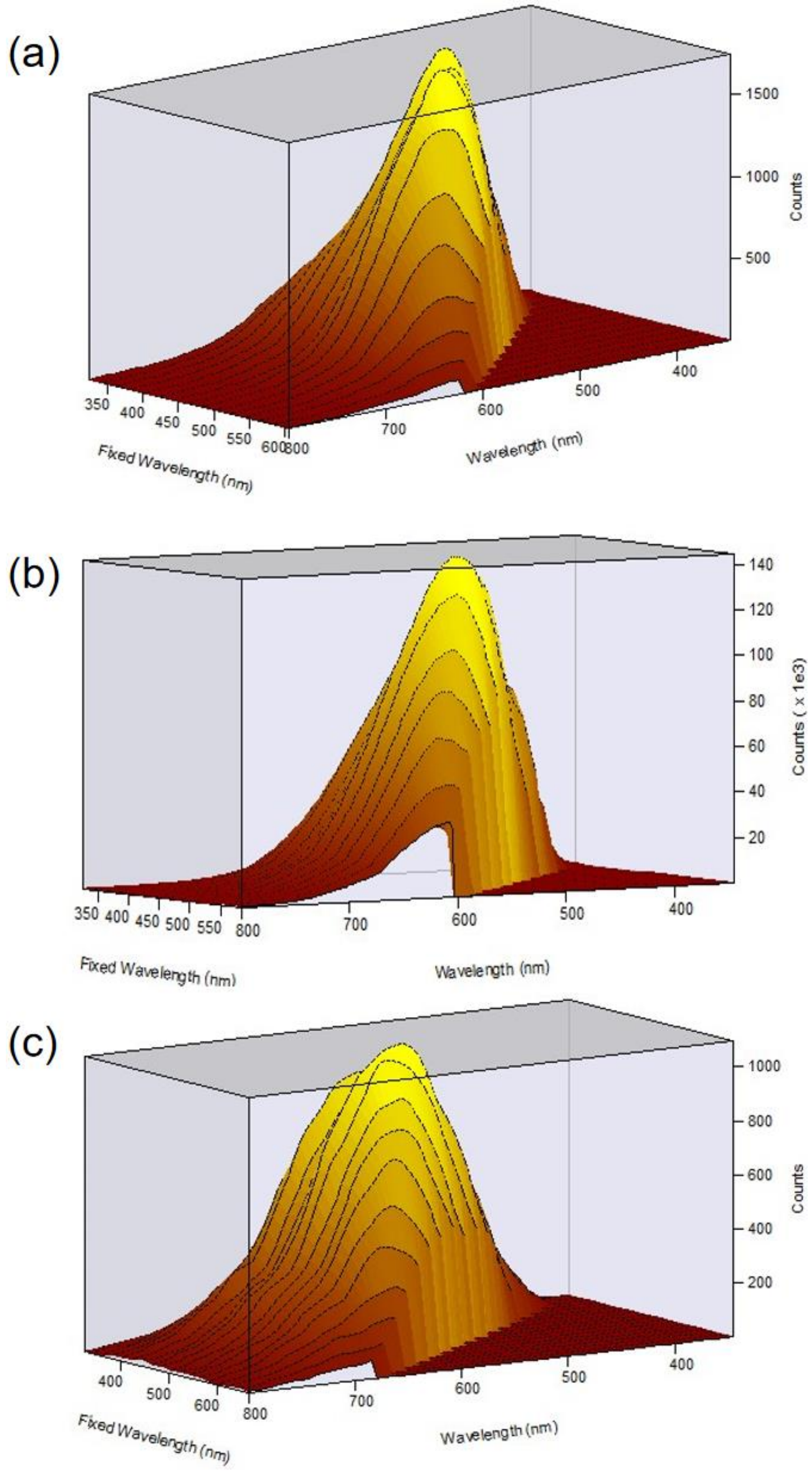

Figure S13. 3D emission spectra of (a) **PUT-a,** (b) **PUT-b** and (c) **PUT-c** powder samples with different excitation wavelengths at room temperature.

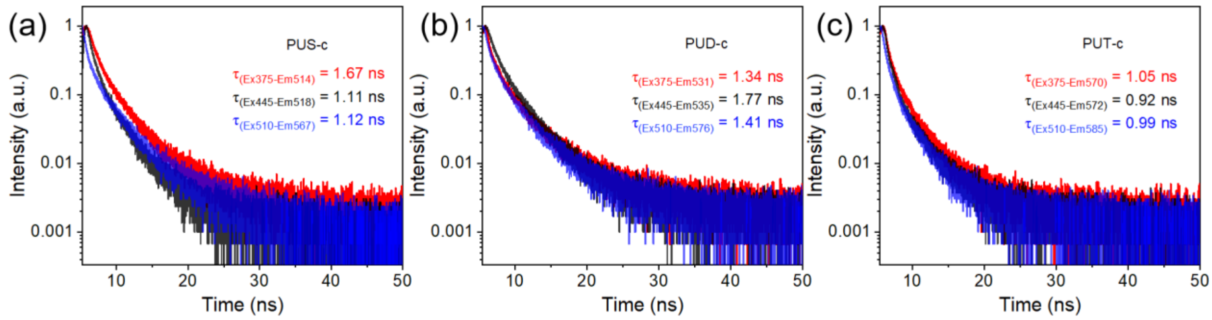

Figure S14. Fluorescence lifetime of **PUS-c**, **PUD-c** and **PUT-c** under different excitation wavelengths.

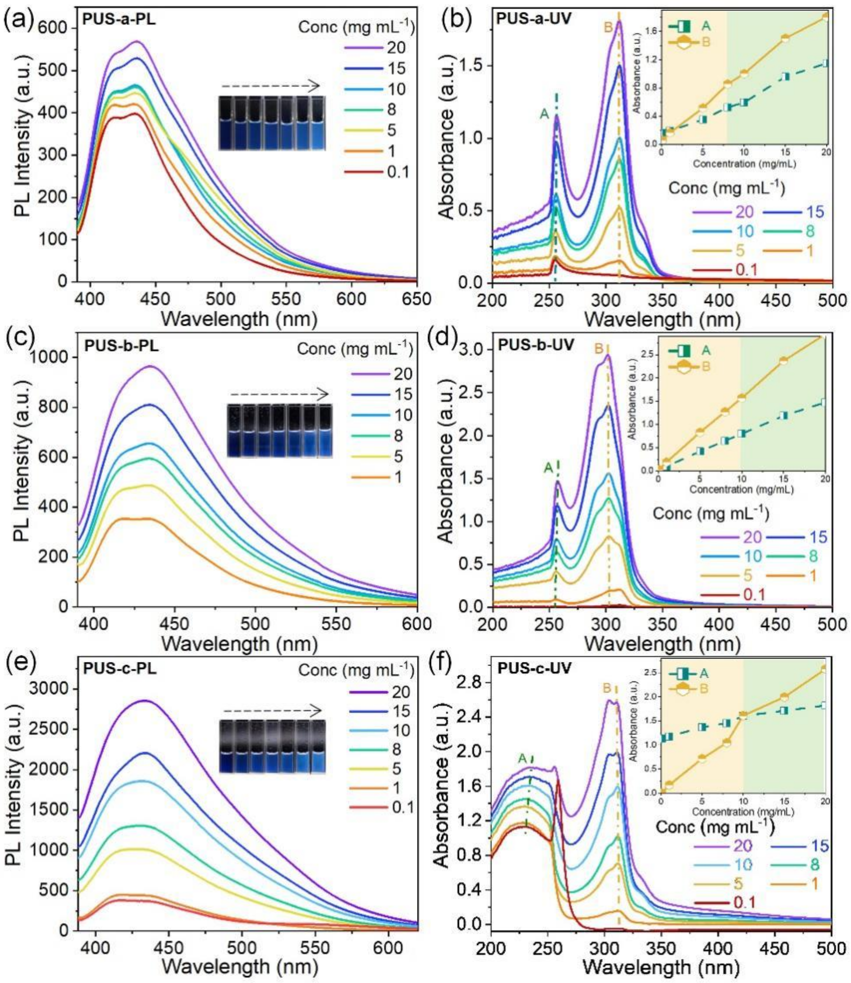

Figure S15. PL spectra of (a) **PUS-a**, (c) **PUS-b**, and (e) **PUS-c**; UV-vis spectra of (b) **PUS-a**, (d) **PUS-b**, and (f) **PUS-c** in DMSO solvent.

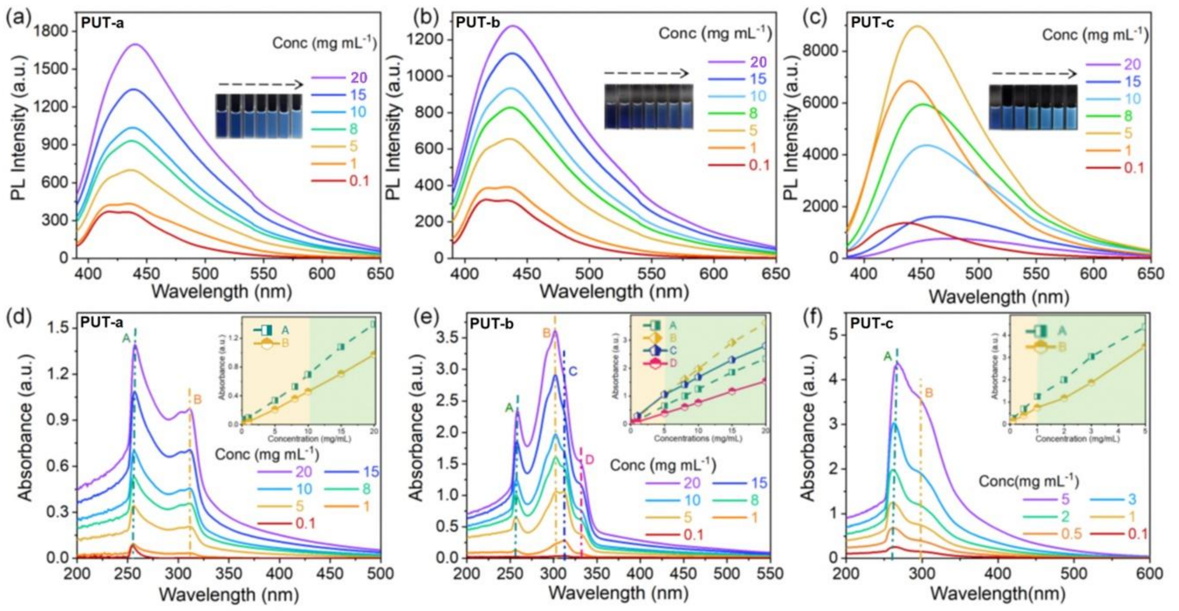

Figure S16. PL spectra of (a) **PUT-a**, (b) **PUT-b**, and (c) **PUT-c**; UV-vis spectra of (d) **PUT-a**, (e) **PUT-b**, and (f) **PUT-c** in DMSO solvent.

4. Theoretical calculations

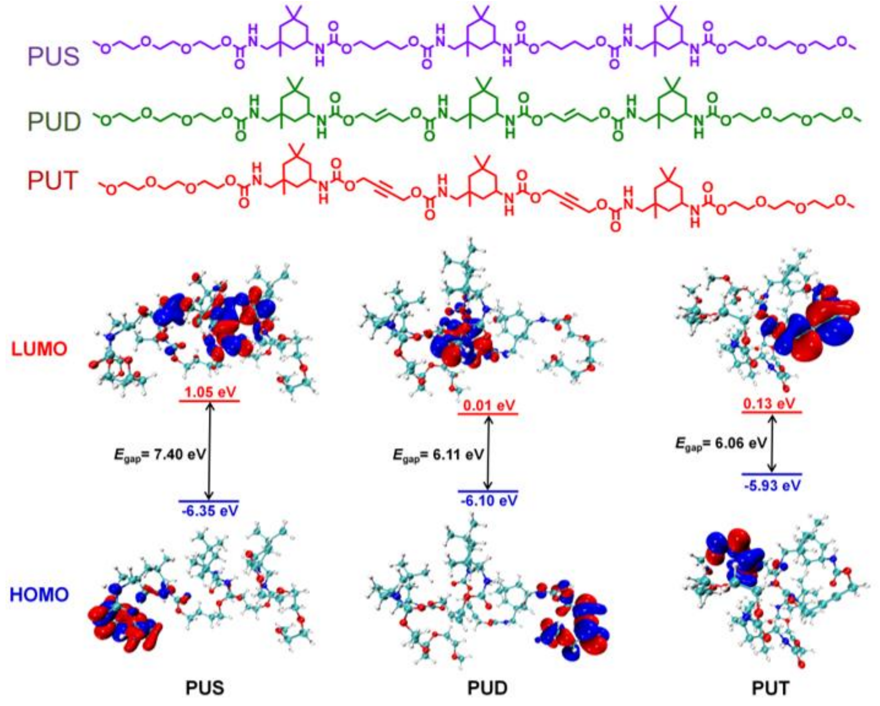

Figure S17. The molecular unit of PUs used for DFT calculations and the frontier molecular orbitals and the HOMO and LUMO energy levels of **PUS**, **PUD** and **PUT**.

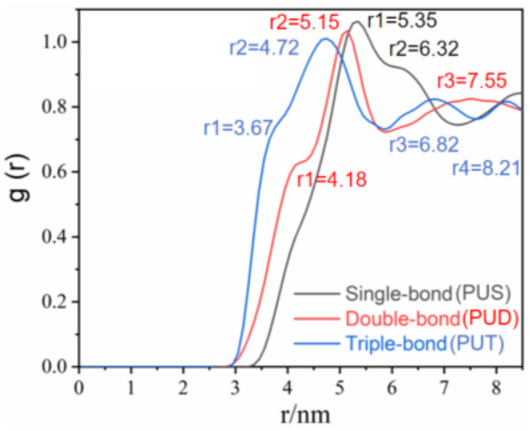

Figure S18. Radial distribution function of single-bond, double-bond and triple-bond in PUs by molecular dynamics simulation.

5. Applications

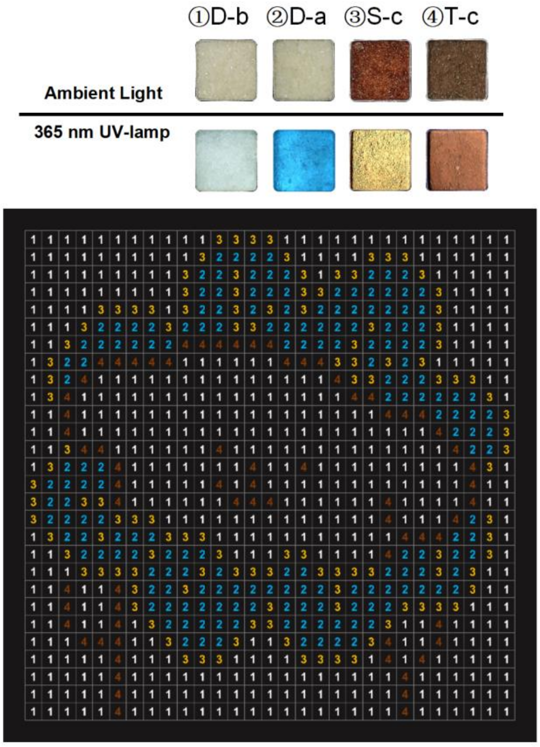

Figure S19. A schematic of the materials (**PUD-b**, **PUD-a**, **PUS-a** and **PUT-c**) and arrangement used in the pixel painting.

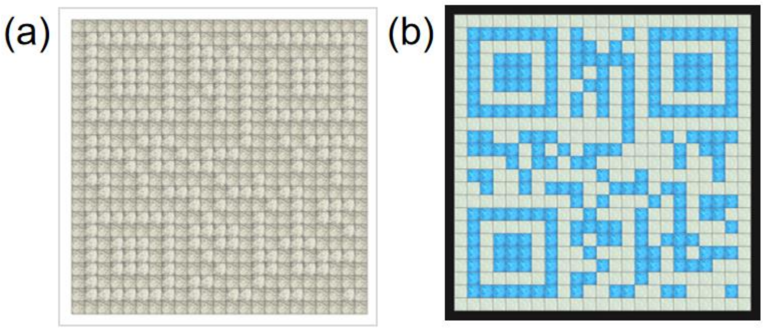

Figure S20. Photographs of a two-dimensional code under (a) daylight and (b) a 365 nm UV lamp.

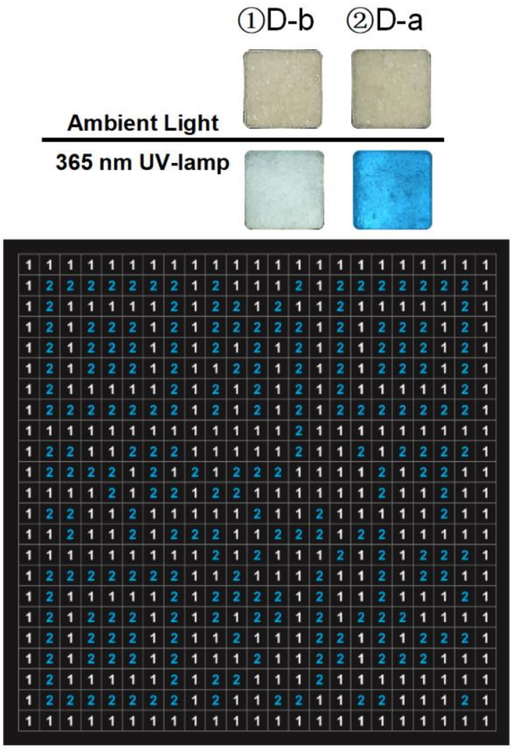

Figure S21. Materials (**PUD-b** and **PUD-a**) and arrangement diagram of the QR code.

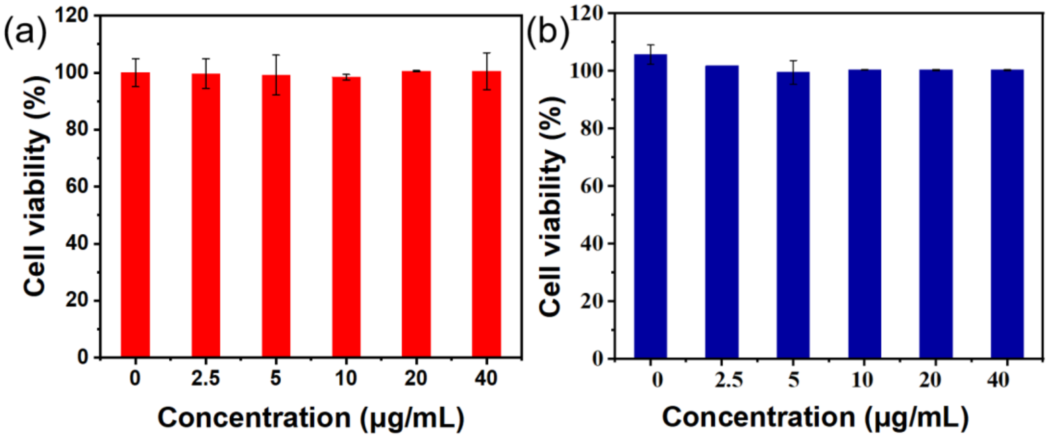

Figure S22. Relative viability of 4T1 cells after 24 h co-incubation with different concentrations of (a) **PUS-b** and (b) **PUT-**c.

Table S1. Molecular weight data of **PUS**, **PUD** and **PUT** from GPC.

	**PUS-a**	**PUS-b**	**PUS-c**	**PUD-a**	**PUD-b**	**PUD-c**	**PUT-a**	**PUT-b**	**PUT-c**
M_n	2874	2740	2119	3870	2481	2190	3668	1328	1282
M_w	4244	3959	2428	5869	3200	2561	4502	1534	1469
M_p	4635	4076	1514	5846	3303	1931	4531	887	877
M_z	5456	5081	2813	7896	4002	3055	5402	1827	1741
**PD**	1.48	1.44	1.15	1.52	1.29	1.17	1.23	1.16	1.15

Table S2. The fluorescence quantum efficiency (QY) and luminescence lifetimes (LT) of **PUS**, **PUD** and **PUT** powder samples at room temperature.

	**PUS-a**	**PUS-b**	**PUS-c**	**PUD-a**	**PUD-b**	**PUD-c**	**PUT-a**	**PUT-b**	**PUT-c**
**QY**	1.4%	1.0%	1.8%	2.5%	2.5%	1.2%	2.0%	1.6%	0.8%
**LT(ns)**[a]	6.53	3.45	2.38	3.36	3.17	3.08	4.11	2.75	1.81
λ_{em}**(nm)**[a]	434	434	528	433	470	552	485	477	575

([a] λ_{ex}=365 nm)

Table S3. Molar absorption coefficient of **PUS**, **PUD** and **PUT** in DMSO solvent (0.0001 mol L^{-1}).

	**PUS-a**	**PUS-b**	**PUS-c**	**PUD-a**	**PUD-b**	**PUD-c**	**PUT-a**	**PUT-b**	**PUT-c**
λ_{abs} (nm)	258	258	264	258	266	288	258	258	320
$\varepsilon(PUs)_{\lambda abs}$ ($L\cdot mol^{-1}cm^{-1}$)	2600	2800	28200	2000	25900	40400	2000	20300	46000

Table S4. Research status of the internal mechanism of colorful non-traditional luminophores.

Literature reference	Proposed mechanism
Angew. Chem. Int. Ed., **2019**, *58*, 3082-3086.	The ring-opening/ring-forming structural transformation of intramolecular B-O bond in response to external stimuli
Angew. Chem. Int. Ed., **2022**, *61*, e202204383.	Rigid-flexible equilibrium effects and spatial coordination bonds promote charge exchange in aggregation bodies
Adv. Opt. Mater., **2023**, *11*, 2300715.	Through-space charge transfer (TSCT)

Macromolecules, **2023**, *56*, 4541-4549.	Aggregation of oxygen atoms due to conformational limitation of polymer
J. Mater. Chem. C, **2024**, *12*, 1040-1046.	Inter/intra-molecular hydrogen bonds and through-space dative bonds
Macromolecules, **2024**, *57*, 3121-3130.	Hydrogen bonding and van der Waals (vdW) interactions
Nat. Commun., **2024**, *15*, 366.	The amine-Polyester complexation process produces enhanced through-space interactions (TSI)
Mater. Horiz., **2024**, *11*, 1579-1587.	Heteroatoms promote spatial conjugation between heteroatoms and carbonyl groups
J. Am. Chem. Soc., **2024**, *146*, 10889-10898.	Spatial conjugation brought about by short contacts; intra-chain/inter-chain charge transfer or TSCT
**This work**	**Through-space n-π interactions and hydrogen bonding**

Table S5. Average counts of hydrogen bond (n_H) and C=O/C=C n-π interaction (n_π) [a] during three-stage MD simulations.

	T_{syn} (K)	n_H, MD1	n_H, MD2	n_H, MD3	n_π, MD1	n_π, MD2	n_π, MD3
**PUS-a**	338.0	145.1	149.8	159.8	482.8	477.7	474.7
**PUS-b**	348.0	129.0	135.5	143.7	474.2	464.3	484.8
**PUS-c**	423.0	144.9	148.3	173.3	481.2	463.1	503.3
**PUD-a**	338.0	147.1	144.2	153.3	488.6	471.5	458.5
**PUD-b**	348.0	150.4	144.2	151.2	494.0	459.6	454.0
**PUD-c**	423.0	149.8	132.2	160.9	481.6	442.6	453.7
**PUT-a**	338.0	136.4	132.5	134.7	568.0	556.0	554.1
**PUT-b**	348.0	124.1	125.6	132.8	573.6	583.0	585.8
**PUT-c**	423.0	144.5	132.2	154.6	596.6	511.9	510.1

[a] Criterion to recognize n-π interaction: distance between midpoints of two bonds smaller than 4.0 Å. For **PUS-a/b/c**, data were collected for C=O and C-C bond at the same position with C=C/C≡C bond in **PUD/T**.

6. References

[S1] Siu, H.; Duhamel, J. Molar Absorbance Coefficient of Pyrene Aggregates in Water Generated by a Poly(Ethylene Oxide) Capped at a Single End with Pyrene. *J. Phys. Chem. B.* **2012**, *116*, 1226-1233.

[S2] Grimme, S. Semiempirical GGA-type Density Functional Constructed with a Long-Range Dispersion Correction. *J. Compu. Chem.* **2006,** *27*, 1787-1799.

[S3] Perdew, J. P.; Burke, K.; Ernzerhof, M. Generalized Gradient Approximation Made Simple. *Phy. Rev. Lett.* **1996,** *77*, 3865-3868.

[S4] Frisch, M. J.; Trucks, G. W.; Schlegel, H. B.; Scuseria, G. E.; Robb, M. A.; Cheeseman, J. R.; Scalmani, G.; Barone, V.; Petersson, G. A.; Nakatsuji, H., et al. *Gaussian 16 Rev. C.01*, Wallingford, CT, 2016.

[S5] Lu, T.; Chen, F. Multiwfn: A Multifunctional Wavefunction Analyzer. *J. Comput. Chem.*

2012, *33*, 580-592.

[S6] Sprenger, K. G.; Jaeger, V. W.; Pfaendtner, J. The General AMBER Force Field (GAFF) Can Accurately Predict Thermodynamic and Transport Properties of Many Ionic Liquids. *J. Phys. Chem. B.* **2015,** *119*, 5882-5895.

[S7] Hess, B.; Kutzner, C.; Van Der Spoel, D.; Lindahl, E. GROMACS 4: Algorithms for Highly Efficient, Load-Balanced, and Scalable Molecular Simulation. *J. Chem. Theory. Comput.* **2008,** *4*, 435-447.

[S8] Tian, L. *Sobtop*, 1.0(dev3.1).

[S9] Darden, T.; York, D.; Pedersen, L. Particle Mesh Ewald: An $N \cdot \log(N)$ Method for Ewald Sums in Large Systems. *J. Chem. Phys.* **1993,** *98*, 10089-10092.

[S10] Bussi, G.; Donadio, D.; Parrinello, M. Canonical Sampling through Velocity Rescaling. *J. Chem. Phys.* **2007,** *126*, 014101.

[S11] Parrinello, M.; Rahman, A. Polymorphic Transitions in Single Crystals: A New Molecular Dynamics Method. *J. Appl. Phys.* **1981,** *52*, 7182-7190.

[S12] Zou, J. Molecular Orbital Kit (MOKIT). https://gitlab.com/jxzou/mokit (accessed 2023.11.01).

[S13] Fales, B. S.; Shu, Y.; Levine, B. G.; Hohenstein, E. G. Complete Active Space Configuration Interaction from State-Averaged Configuration Interaction Singles Natural Orbitals: Analytic First Derivatives and Derivative Coupling Vectors. *J. Chem. Phys.* **2017,** *147*, 094104.

[S14] Sun, Q.; Berkelbach, T. C.; Blunt, N. S.; Booth, G. H.; Guo, S.; Li, Z.; Liu, J.; McClain, J. D.; Sayfutyarova, E. R.; Sharma, S., et al. PySCF: the Python-Based Simulations of Chemistry Framework. *Wires. Comput. Mol. Sci.* **2018,** *8*, e1340.

[S15] Noorpour, M.; Tarighat, A. Molecular Dynamics Study of a Macromolecular Model of Hydrous Sodium Aluminosilicate Geopolymer with Charge Balancing by Extra-Structural Aluminum for Prediction of its Properties. *Ceram. Int.* **2021**, *47*, 19304-19314.

[S16] Wang, R.; Wang, J.; Dong, T.; Ouyang, G. Structural and Mechanical Properties of Geopolymers Made of Aluminosilicate Powder with Different SiO_2/Al_2O_3 Ratio: Molecular Dynamics Simulation and Microstructural Experimental Study. *Constr. Build Mater.* **2020**, *240*, 117935.
